# Exploring bioactive peptides from Andean grains and their potential applications: a review

**DOI:** 10.3389/fnut.2026.1875238

**Published:** 2026-08-05

**Authors:** Beatríz Damiano-Vásquez, Atma-Sol Bustos, Ritva Repo-Carrasco-Valencia

**Affiliations:** 1 Programa de Doctorado en Ingeniería Agroindustrial mención Transformación Avanzada de Granos y Tubérculos Andinos, Universidad Nacional del Santa, Nuevo Chimbote, Ancash, Peru; 2 Instituto de Investigaciones Químicas (IIQ), Universidad Mayor de San Andrés (UMSA), La Paz, Bolivia; 3 Centro de Investigación e Innovación en Productos Derivados de Cultivos Andinos (CIINCA), Universidad Nacional Agraria La Molina-UNALM, Lima, Peru

**Keywords:** amaranth, *Amaranthus caudatus*, cañihua, *Chenopodium pallidicaule*, *Chenopodium quinoa*, functional foods, lupin, *Lupinus mutabilis*

## Abstract

Andean grains, such as quinoa, amaranth, cañihua and lupin, have exceptional nutritional value and remain an underexplored source of bioactive peptides with diverse health-promoting properties. This review analyses 87 studies published between 2015 and 2026, following PRISMA guidelines, to map recent technological and biological advances in bioactive peptide research. Integrating *in vitro*, *in silico*, and *in vivo* evidence, it provides a comprehensive overview of peptide generation, characterization, and biological activities. Advances in ultrafiltration, electrodialysis, chromatography, and high-resolution mass spectrometry have facilitated the separation and identification of peptides exhibiting antioxidant, antihypertensive, antidiabetic, anti-inflammatory, anticancer, antimicrobial, antifatigue and mineral-chelating activities. In silico analysis, particularly molecular docking studies, have revealed strong binding affinities to regulatory proteins involved in metabolic and cellular defence pathways, while animal models have shown beneficial effects partially mediated through gut microbiota modulation. The novelty of this review lies in its multiscale integration of peptide research and its emphasis on underrepresented Andean grains. It highlights emerging technologies such as high hydrostatic pressure, ultrasound, and fermentation as promising strategies to enhance peptide yield and functionality and explore the food formulation that includes bioactive peptides. Overall, this review underscores the potential of Andean grains as sustainable sources of next-generation ingredients for a wide range of applications across the food, health, and technology sectors.

## 1 Introduction

Andean grains have been consumed since pre-Inca times. Currently, these foods are valued due to their nutritional and functional properties (1). They are considered superfoods and gluten-free, which makes them a substitute for conventional cereals (2). They also adapt to unfavorable climatic conditions and are the main source of income for economically disadvantaged farmers.

The main Andean grains are quinoa (*Chenopodium quinoa*), cañihua (*Chenopodium pallidicaule)*, kiwicha or amaranth (*Amaranthus caudatus)* and Andean lupin, regionally known as *tarwi*, *tarhui* or *chocho* (*Lupinus mutabilis*). These grains are considered to be native to the Andes (3).

Quinoa is a rich source of high-quality protein (12–23%), containing all essential amino acids, particularly lysine (5.1–6.4%) and methionine (0.4–1.0%) (4–6). It also contains bioactive compounds such as polyphenols, phytosterols, flavonoids and carotenoids (7), and is rich in vitamins, minerals, unsaturated fatty acids and oleic acid (8). Furthermore, it has a low glycaemic index making it a suitable option for glycemic control (9–12).

Amaranth is a good source of protein (13.5–15.88%) (13, 14) and has an excellent balance of essential amino acids (15), particularly lysine, tryptophan, and methionine (13). It is also rich in flavonoids and other bioactive compounds, such as phenolic acids, anthocyanins, tannins, and phytosterols (15).

Cañihua, despite having received limited research attention, contains protein (15–19%) and a broad spectrum of amino acids, including high levels of lysine and other released during digestion like isoleucine and threonine (16); Unlike other Andean seeds (quinoa and amaranth), its low saponin content gives it a non-bitter taste (17). It also provides bioactive compounds such as fiber (18), flavonoids and other phenolic compounds (19).

The Andean lupin is a legume native to the Andes and is the only domesticated lupin species from South America. It is part of a large genus with over 300 species, but only a few are cultivated, including *L. mutabilis*, *L. albus*, *L. angustifolius*, and *L. luteus*. *Lupinus mutabilis* is the primary Andean lupin species cultivated for food and agriculture. These legumes are characterized by their high content of proteins (32–48%), fiber, lipids, polyphenols and oligosaccharides (20, 21). Leucine and lysine are the most abundant essential amino acids while glutamic acid, arginine and aspartic acid are the predominant non-essential amino acids (22). Additionally, they contain high levels of oil and fiber, low levels of carbohydrate, and high concentration of alkaloid (23). Therefore, its consumption is limited due to its bitterness, related to the presence of quinolizidine alkaloids, which are considered antinutrients (24, 25).

In recent years, the study of bioactive peptides derived from food matrices has gained considerable importance. Foods contain encrypted peptide sequences within their protein structures with bioactive properties, which are generated or released from proteins through proteolysis, during food processing or digestion. These protein fragments can improve human health and prevent metabolic diseases, inflammation, hypertension, obesity and diabetes mellitus (26, 27). The biological activity of peptides depends on their structure, the characteristics of the constituent amino acids (functional groups, size, charge and hydrophobicity) and the sequence of the amino acids (position of amino acids) within the peptide chain (28). By altering the structure of native peptides through processes such as hydrolysis, fragments with specific biological functions relevant to health and industry can be released (29).

Andean grains are rich sources of protein and contain bioactive peptides capable of modulating key physiological processes with positive health effects. Peptides derived from quinoa, amaranth, cañihua, and lupin have demonstrated antioxidant activity (7, 11), antihypertensive effects through ACE inhibition (30, 31), antidiabetic activity via DPP-IV, *α*-amylase, and α-glucosidase inhibition (32, 33), as well as anti-inflammatory effects mediated by NF-κB suppression (34). In addition, anticancer activity has been observed in colorectal cancer models (25, 35), antimicrobial properties against Gram-positive and Gram-negative bacteria and fungi (17, 36), anti-hemolytic protection of erythrocytes (37), and antifatigue effects in exercise models (38). These multifunctional properties highlight the potential of Andean grains as valuable raw materials for the development of functional foods and nutraceuticals.

This review provides a comprehensive and critical synthesis of the recent advances in the field of bioactive peptides derived from Andean grains, based on a structured literature search conducted in accordance with the PRISMA guidelines. While quinoa accounts for the majority of the available evidence, the review also examines recent findings on amaranth, cañihua and lupin. The review critically discusses strategies for generating peptides, techniques for purifying and identifying them, computational approaches for characterizing them and predicting their bioactivity, their reported biological activities, and their emerging applications in food systems. Integrating evidence from *in vitro*, in silico and *in vivo* studies, the review identifies knowledge gaps, highlights underrepresented Andean crops and outlines future research priorities to facilitate translation of bioactive peptides into industrial applications and clinically relevant functional foods.

## 2 Search strategy, filtering, article selection and data processing

**Figure 1:**
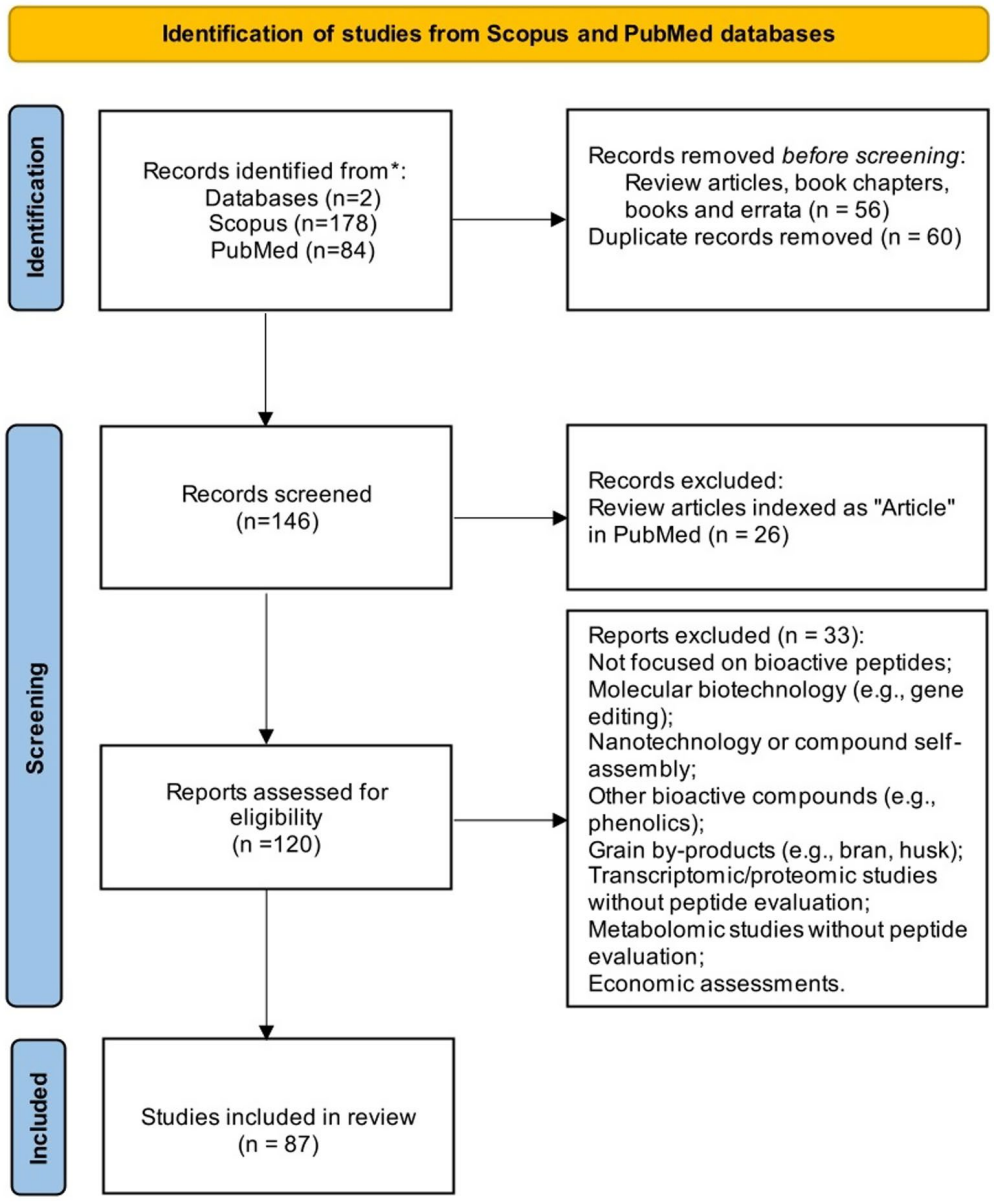
PRISMA 2020 flow diagram of the study selection process.

A comprehensive literature search was conducted in the Scopus and PubMed databases to identify studies on bioactive peptides derived from Andean grains. The initial search was performed on 28 October 2024 and updated on 16 June 2026. The search terms included the names of Andean grains (quinoa, *Chenopodium quinoa*, tarwi, chocho, *Lupinus mutabilis*, cañihua, kañiwa, *Chenopodium pallidicaule* and *Amaranthus caudatus*) combined with keywords related to peptide bioactivities, including bioactive, anti-hypertensive, antidiabetic, anti-inflammatory, antioxidant, antimicrobial, antithrombotic, anti-hyperlipidemic and anti-haemolytic peptides. To focus on the target Andean grain species, studies involving non-target species such as *Lupinus albus*, *Lupinus angustifolius*, *Lupinus luteus*, *Amaranthus hypochondriacus*, *Amaranthus cruentus*, *Amaranthus retroflexus* and *Amaranthus mantegazzianus* were excluded. Only peer-reviewed original research articles published in English between January 2015 and 16 June 2026 were considered. The search retrieved 262 records. After the removal of duplicate records and publication types that did not meet the eligibility criteria (review articles, book chapters, books and errata), 146 records were screened according to the PRISMA 2020 guidelines (39). Subsequently, 120 full-text articles were assessed for eligibility. To improve the methodological consistency of the evidence synthesis, all eligible full-text articles underwent a methodological quality assessment based on the following predefined criteria: (i) a clear description of the Andean grain source and protein substrate; (ii) a detailed description of the peptide generation strategy (e.g., enzymatic hydrolysis, fermentation, germination, emerging technologies, or simulated gastrointestinal digestion); (iii) appropriate methods for peptide characterization; (iv) clearly described protocols for bioactivity assessment; and (v) quantitative reporting of outcomes relevant to the study objectives (e.g., inhibitory activity, IC₅₀ values, degree of hydrolysis, antioxidant capacity, peptide identification, or other bioactivity-related parameters). Studies lacking sufficient methodological information or that were not directly related to bioactive peptides from Andean grains were excluded. Finally, 87 studies were included in the narrative review. A formal meta-analysis or risk-of-bias assessment was not considered appropriate given the methodological heterogeneity of the included *in vitro*, *in silico*, *in vivo* and food application studies. The study selection process is summarized in Figure 1.

## 3 Generation of bioactive peptides from Andean grains

Peptides can be generated through physical, chemical and biological processes. In these processes, native proteins are cleaved into short amino acid sequences, typically 2–20 residues long, which are classified as peptides. These processes may induce structural modifications in proteins, such as changes to the secondary and tertiary structures, thereby increasing the accessibility of cleave sites and influencing the generation of peptides with different biological (40). In food systems, bioactive peptides can be generated through a variety of technological strategies, as summarized in Step 1 of Figure 1, such as enzymatic hydrolysis, fermentation, chemical hydrolysis and gastrointestinal digestion have been reported as peptide-releasing strategies (41–43). More recently, emerging technologies, including ultrasound, microwave treatment, high hydrostatic pressure, pulsed electric fields, ohmic heating, and critical water processing have been investigated to enhance protein accessibility and intensify peptide generation (41, 44). Other advanced approaches, such as chemical peptide synthesis, recombinant DNA-based biosynthesis, and *in silico* design strategies have also been reported as new trends (41, 45–47). However, the effectiveness of these strategies as reported varies considerably due to differences in grain characteristics, processing conditions and enzyme specificity. This makes direct comparisons between studies difficult.

Accordingly, this section introduces the main peptide-generation strategies that have been reported and applied to Andean grains, including enzymatic hydrolysis, germination, fermentation, emerging technologies, and simulated gastrointestinal digestion. Representative studies are summarized in Table 1, whereas an extended compilation of studies is provided in Supplementary Table S1.

**Table 1:** Strategies to generation of bioactive peptides from Andean grains.

Andean grain	Protein source	Generation strategy	Enzyme/process	Experimental conditions	Main results	Reference
Quinoa	Protein isolate	Enzymatic Hydrolysis	Chymotrypsin; protease; bromelain	4% (w/v); E: S ratio 1:100 (w/w); pH 7.0–8.0; 6 h	DH: chymotrypsin (66.05%) > protease (63.3%) > bromelain (51.91%) after 6 h	(58)
Quinoa (Pasankalla)	Protein concentrates; globulins	Enzymatic Hydrolysis	Thermolysin	2.5% (w/v); E: S ratio 1:100 (w/w); pH 8.0; 50 °C; 4–24 h	DH: concentrate (25.7 –35.4%) at 4 to 24 h; globulins (20.2–21.8%); *in silico digestion* predicted peptides <1,000 Da predicted	(11)
Kiwicha (*A. caudatus*)	Protein isolate	Enzymatic Hydrolysis	Bioprotease LA-660	5% (w/v); pH 8.0; 50 °C; 60 min	DH: rapid hydrolysis in first 5 min; maximum 32.02% at 60 min	(14)
Quinoa	Protein concentrate	Enzymatic Hydrolysis	Alcalase	1% (w/v) protein; E: S ratio 10 μL/100 mg protein solution; pH 8.0; 50 °C; 30 min	DH: Reached 27%. The hydrolysate showed immunomodulatory activity (cytokine release)	(52)
Quinoa	Protein (Flour)	Enzymatic Hydrolysis	Papain → Trypsin	5% (w/v) protein; Papain: E: S 1%; trypsin: 1% (w/w); pH 7.5; 50 °C; 5 h	DH: associated with enrichment in hydrophobic amino acids (60.79%)	(137)
Lupin	Protein concentrate	Enzymatic Hydrolysis (RSM Optimization)	Alcalase	4% (w/v); pH 8.5; 50 °C; Optimization: E/S: ratio 0.19–2.41%; time 35–205 min	DH: predicted maximum 45.7% (E/S 1.72%, 133 min); experimental DH 45.1%	(23)
Lupin	Protein concentrate	Enzymatic Hydrolysis	Alcalase → Neutrase	2.5% (w/v); 50 °C; Alcalase: pH 8.3, 0.385 AU/g, 60 min; Neutrase: pH 7.0, 50 LAPU/g, 120 min	Peptide size: low MW (<3,000 Da); short peptides (6–9 residues) identified	(103)
Amaranth	Fractions (albumin, globulin, glutelin)	Enzymatic Hydrolysis	Alcalase → Trypsin	Alcalase: pH 10, 37 °C, 20 min; Trypsin: pH 8, 37 °C, 20 min	DH: ~25.8–25.9% (glutelins/isolate); globulin-P showed lowest DH (10.6%)	(63)
Cañihua (Cupis)	Protein concentrate	Enzymatic Hydrolysis (Single/sequential)	Alcalase Neutrase Flavourzyme	2.5% (w/v); 50 °C; 240 min total. Enzyme 1: (0–120 min) Enzyme 2: (120–240 min) pH: (alcalase (8.3), Flavourzyme and Neutrase (7.0)	DH: Alcalase-Flavourzyme reached 60.2% Single enzymes: Alcalase (44.9%), Neutrase (35.9%), Flavourzyme (9.6%)	(19)
Quinoa	Protein	Enzymatic Hydrolysis (Single/sequential)	Alcaline protease; Trypsin; Alcaline protease → Trypsin	5% (w/v); 40 °C; 4,000 U/g enzyme Optimal: Simultaneous addition (1:1 ratio) for 4 h	DH: simultaneous dual-enzyme hydrolysis showed highest DH (28.41%) vs. single or sequential treatments	(66)
Quinoa	Flour	Fermentation	*Lactobacillus paracasei* CICC 20241	Solid-state fermentation; purification by ultrafiltration (<3,000 Da)	Peptide profile: 91 peptides identified; fermentation hydrolyzed proteins into peptides <3,000 Da	(76)
Quinoa	Protein isolate	HPH Pretreatment + Enzymatic Hydrolysis	HPH + Alcalase	HHP: 50, 100, 180 MPa (1 cycle), inlet 22.1 °C; Hydrolysis: 5% (w/v), pH 8.0, 50 °C, 4 h	DH: 13.53% (50 MPa); 15.99% (100 MPa); control 15.98%; 50 MPa reduced DH but generated more potent ACE-inhibitory peptides	(82)
Quinoa	Protein concentrate	Hydrolysis + EDUF	Alcalase + EDUF	Hydrolysis: 5% (w/v); Alcalase: 0.092 mL (2.4 U/g protein); pH 8.0, 50 °C	DH: 23%; EDUF fractions enriched peptides 0.4–5,000 Da; anionic (1.2%) and cationic (3%) fractions recovered	(32)
Quinoa	Flour	Malting + SGID	Malting (Endogenous) + Pepsin/Trypsin	Malting: 8–15 °C, 44–52% moisture, 3–7 d; Digestion: INFOGEST	Peptide profile: oligopeptides <20 kDa; DPP-IV inhibitory peptides identified (AFP, HI, HL, RI, RL, IR, LR)	(72)
Amaranth and Cañihua	Protein concentrate	SGID	Pepsin → Pancreatin	INFOGEST: gastric (pH 3, 2 h); intestinal (pH 7, 2 h)	Digestibility: amaranth 79.19%; cañihua 71.22%	(90)

ACE, angiotensin-converting enzyme; AU, Anson units; DH, degree of hydrolysis; DPP-IV, dipeptidyl peptidase IV; EDUF, electrodialysis with ultrafiltration membranes; E:S, enzyme-to-substrate ratio; HPH, high-pressure homogenization; LAPU, leucine aminopeptidase units; MW, molecular weight; RSM, response surface methodology; SGID, simulated gastrointestinal digestion.

### 3.1 Enzymatic hydrolysis

Enzymatic hydrolysis is the primary method used to obtain bioactive peptides from food protein matrices. Proteolytic enzymes selectively cleave peptide bonds, releasing short-chain peptides with potential biological activity, and avoiding the use of organic solvents or toxic reagents (48). The efficiency of the process is determined by the specificity of the enzymes used, the hydrolysis conditions (pH, temperature and enzyme-to-substrate ratio) and the structural characteristics of the protein source (49, 50).

The low solubility and compact structure of native quinoa proteins limit their functionality. However, controlled enzymatic hydrolysis can improve technofunctional properties such as solubility, emulsification and foaming capacity (12), while enhancing bioactivity (32, 49). As shown in Table 1, quinoa protein concentrates, isolates and fractions have been hydrolyzed using a wide range of proteases. Alcalase is the most extensively studied enzyme, yielding degrees of hydrolysis (DH) ranging from 9.87 to 61.8%, depending on the technologies used or the hydrolysis strategies employed (7, 13, 32, 33, 51–55). Alcalase hydrolysates were enriched in low-molecular-weight peptides, with up to 87.4% below 2000 Da, mainly within the 200–2000 Da range (49). As a serine endopeptidase, alcalase efficiently releases bioactive peptides (56). Coupling alcalase hydrolysis with electrodialysis and ultrafiltration results in DH values of around 23% and peptide fractions of between 400 and 1,500 Da (31, 32).

Sequential hydrolysis further increases the efficiency with which peptides are generated in quinoa. The alcalase–flavourzyme combination achieved DH values of up to 61.8% (13), which reflects the complementary action of endo- and exopeptidases. Alcalase increases the availability of terminal sites, which flavourzyme subsequently cleaves, yielding shorter peptides and free amino acids (50, 57). Therefore, alcalase generated uniform and large peptides (53).

Other enzymes exhibited variable hydrolytic performance in quinoa. For example, chymotrypsin achieved DH values of 15.8–88.95%, bromelain 34.99–76.10%, pronase E up to 82.52%, protease up to 63.3%, and trypsin 17.9–50.2% (37, 58, 59). Pepsin, pancreatin, neutrase, thermolysin and compound protease generally produced moderate DH values (~ 14–36%), depending on the protein fraction and the reaction conditions (11, 12, 49). By contrast, Flavourzyme and neutral protease produced low DH values (<10%) when used alone, while papain demonstrated similar limited efficiency (5–15%), despite increasing free amino groups and modifying SDS-PAGE profiles (24, 60, 61). Sequential pepsin–trypsin hydrolysis of germinated quinoa yielded a DH value of 27.27% (61). Overall, DH increased with hydrolysis time as high-molecular-weight peptides were progressively cleaved (37).

In amaranth, the bromelain and Bioprotease LA-660 treatments produced DH values of approximately 47.2 and 32.02%, respectively (14, 37). As with quinoa, the alcalase–flavourzyme combination markedly enhanced hydrolysis, reaching 76.3% DH within 240 min compared to 19.5% with Flavourzyme alone (13). Moderate DH values of 29.2% were reported for *Amaranthus hypochondriacus* proteins hydrolyzed with Alcalase, with globulin-rich fractions showing greater resistance to hydrolysis than glutelins and isolates (62, 63).

In lupin, hydrolysis using purified *Pseudomonas* sp. proteases and commercial alcalase resulted in DH values of approximately 22 and 15%, respectively. Alcalase promoted faster breakdown of proteins into low-molecular-weight peptides (<10 kDa), whereas *Pseudomonas* sp. proteases generated larger peptide fractions (up to ~14 kDa) (64). Optimizing the process significantly improved hydrolysis efficiency, with optimized alcalase treatment (E/S 1.72%, 133 min) increasing DH to 45.1%, which correlated with enhanced radical scavenging activity (23).

In cañihua, alcalase hydrolysis produced DH values ranging from 13 to 54% across the albumin, 7S globulin, 11S globulin and glutelin fractions (17). While research in this area is limited, alcalase (44.9% of DH) has been shown to outperform neutrase and flavourzyme in single-enzyme systems. Furthermore, the alcalase–flavourzyme combination has been found to exceed 50% of DH under optimized conditions (19).

Protein purity is also critical for evaluating hydrolysis performance. Fourier-transform infrared spectroscopy analyses suggest that hydrolysis and purification induce conformational changes that expose cleavage sites and boost antioxidant capacity (65, 66). Overall, variability in DH reflects differences in enzyme specificity and substrate affinity (64), while peptide solubility is influenced by enzyme type and concentration, molecular weight, surface charge, temperature and pH (49).

### 3.2 Germination

Germination is a low-cost biological process that activates endogenous endopeptidases and initiates the controlled degradation of storage proteins, such as 11S globulins and 2S albumins of quinoa (67). During this process, enzymatic activity induces structural and biochemical modifications in proteins, including conformational rearrangements that make them more susceptible to subsequent proteolytic cleavage. These changes support the mobilization of nutrients for seedling growth, while simultaneously modifying the catalytic and binding sites within the protein matrix (68, 69).

Beyond its physiological role, germination enhances the nutritional quality of grains by improving protein digestibility, reduces antinutritional factors (70). This process is also associated with the release and accumulation of free amino acids, which can reach a concentration of ~672 mg/g of protein after 12 h of germination and after digestion (67), alongside an increase in total amino acid content (67, 71). These changes create a more favorable substrate for subsequent enzymatic hydrolysis and peptide generation.

Electrophoretic profiles showing the appearance of low-molecular weight bands reflect evidence of peptide formation during germination, with the intensity and distribution of these bands depending on quinoa variety and germination conditions (61, 69). Specifically, the acidic and basic subunits of 11S globulin (32000–39,000 Da) of quinoa undergo significant degradation after 36–48 h of germination. However, prolonged germination periods (36–72 h) may promote the reorganization of proteins into more compact structures, thereby reducing enzymatic accessibility and limiting further hydrolysis (67).

The facilitating role of germination becomes more evident when combined with exogenous proteolytic enzymes. The hydrolysis of germinated quinoa using neutrase, alcalase, flavourzyme or papain resulted in degrees of hydrolysis ranging from 5.10 to 23.45% (61). Similarly, germination followed by gastrointestinal digestion significantly enhances peptide release. For instance, the DH achieved through pepsin–trypsin digestion of germinated quinoa was as high as 27.27%, compared to 8.7% for non-germinated samples (61), and 90% of the resulting peptides were less than 1,000 Da in size (67). This could indicate that germination prepares proteins for more efficient digestion. However, these results also suggest that greater intensity of germination does not necessarily lead to greater hydrolysis, which highlights the complexity of endogenous enzymatic dynamics.

Malting, a controlled and optimized form of quinoa germination, further illustrates the importance of processing conditions. Kröber et al. (72) demonstrated that moisture, temperature and time have a strong influence on peptide profiles, leading to the formation of oligopeptides (< 20,000 Da) and, following simulated gastrointestinal digestion, specific di- and tripeptides associated with improved DPP-IV inhibitory activity. Optimal conditions (52% moisture, 15 °C, 3 days) maximized these functional outcomes (72).

Overall, germination should be considered a strategy that facilitates, rather than generates, peptides, enhancing the efficiency of subsequent enzymatic hydrolysis and gastrointestinal digestion. However, research in this area is still in its initial stage, particularly with regard to Andean grains and quinoa varieties. Factors such as non-protein components in isolates and heat-induced protein compaction may reduce hydrolysis efficiency (24, 73). Therefore, future research should focus on optimizing germination and malting protocols, systematically linking processing parameters with peptide profiles.

### 3.3 Fermentation

Fermentation produces bioactive peptides through microbial proteolysis, whereby secreted enzymes break down proteins into low-molecular-weight peptides and free amino acids, or generate *de novo* sequences with potential bioactivity, thereby reducing antinutritional content. The resulting peptide profile depends on factors such as the microbial strain, fermentation type and processing parameters, including time, temperature and substrate (74–78). Both solid-state and submerged systems are used, with solid-state systems generally offering lower contamination risks (79). Although research on quinoa remains limited, the available evidence is promising. For example, solid-state fermentation with *Lactobacillus paracasei* CICC 20241 increased antihypertensive activity from 52.06 to 86.50%, which has been linked to enhanced peptide release (76).

Regarding fungal fermentation, *Pleurotus ostreatus* reduced peptides >12.5 kDa from 90% to 65–85% and enriched smaller peptides from 15 to 35%. However, hot-air drying (70 °C) partially reversed the amino acids content reduction caused by fermentation, potentially due to Maillard reactions, whereas freeze-drying showed no clear mechanism (78). The amino acid composition was also altered, with notable increases in valine, isoleucine, leucine, and tryptophan in black quinoa. Similarly, *Rhizopus oligosporus* ATCC 64063 increased peptide levels by up to 15-fold in black quinoa and by up to 8-fold in red quinoa, enriching hydrophobic and aromatic residues and enhancing antioxidant potential (80).

Further studies have confirmed the role of native lactic acid bacteria. Rizzello et al. (77) isolated 26 strains from quinoa and used them as starter cultures. After 24 h, the concentration of peptides increased from 15.08 to 20.92 mg/mL. Among these strains, *Lactobacillus plantarum* T0A10 exhibited functional inhibition of linoleic acid oxidation, suggesting its potential application in functional foods. More recently, Zhu et al. (36) reported that combining enzymatic hydrolysis (lipase and amylase) with mixed fermentation increased the proportion of 1000 Da peptides from 1.7 to 30.3%, thereby enhancing antimicrobial activity.

In general, fermented quinoa emerges as a promising source of bioactive peptides, particularly when fermentation is combined with strategies such as enzymatic hydrolysis or drying. Studies have reported consistent improvements in peptide release, molecular weight distribution and functional activities. However, current research is largely restricted to quinoa, and the generation of fermentation-driven peptides in other Andean grains, such as amaranth, cañihua, and lupin, has not been reported.

### 3.4 Emerging technologies

Conventional enzymatic hydrolysis is widely used but can be limited by the specificity of enzymes and the structural resistance of proteins; enzymes only cleave at defined sites (51). Alternative technologies that have been investigated to overcome these limitations include high hydrostatic pressure (HHP), high-pressure homogenization (HPH), high-intense ultrasound (HIU) and extrusion.

High hydrostatic pressure involves immersing the product in water and applying a pressure of 100–1,000 MPa at a temperature of 0–120 °C (81). In quinoa, treatments at 200–600 MPa combined with alcalase have been shown to enhance hydrolysis and peptide release. The best performance was achieved at 300 MPa, yielding 1.3 times more peptides than untreated samples. These improvements have been attributed to protein unfolding and greater accessibility of cleavage sites. However, higher pressures may promote the formation of soluble aggregates (51).

The impact of high-pressure homogenization has also been evaluated. De Carvalho Oliveira et al. (82) reported that pretreatment of quinoa protein at 50 MPa reduced the degree of hydrolysis compared to the control and samples treated at higher pressures (100–180 MPa). This reduction could attribute to protein unfolding, followed by re-aggregation, which limited access to the alcalase enzyme. However, despite the lower degree of hydrolysis, the hydrolysate at 50 MPa exhibited conditions that favored the release of low-molecular-weight peptides (< 5,000 Da) and promoted the degradation of globulins (>25 kDa). This resultant hydrolysate exhibits higher bioactivity with antihypertensive and antioxidant properties.

In quinoa, high-intensity ultrasound (20 kHz) at 400 W for 20 min was associated with enhanced alcalase hydrolysis, achieving a degree of hydrolysis of around 24%. This effect was linked to ultrasound-induced alterations in secondary and tertiary structures. Ultrasound treatment significantly decreased *α*-helix content and increased random coil structures, indicating partial unfolding of the secondary structure of protein. Changes in tertiary structure were evidenced by increased exposure of hydrophobic groups. However, excessive ultrasound intensity (600 W) reduced hydrolysis efficiency, likely due to protein aggregation (83).

In amaranth, extrusion induced partial protein denaturation, reducing molecular weight and facilitating enzymatic accessibility. Montoya-Rodríguez et al. (84) observed that this processing significantly accelerated peptide release. Specifically, after 10 min of hydrolysis with pancreatin, around 28% of the peptides in the extruded samples were smaller than 1,000 Da, compared to ~10% in the untreated amaranth. Also, the combination of extrusion and germination in quinoa increased the efficiency of alcalase hydrolysis, suggesting that these pre-treatments improve protein accessibility and accelerate peptide release (85). Compared with conventional hydrolysis, the available evidence suggests that these emerging technologies may enhance peptide release and modulate molecular weight distribution. This supports the potential of quinoa and amaranth as sources of functional protein ingredients. However, variability in experimental conditions and the limited number of studies available restrict direct comparisons between studies, emphasizing the need to further optimize and standardize processing parameters.

### 3.5 Generation of peptides through *in vitro* simulation of gastrointestinal digestion

Simulated gastrointestinal digestion (SGID) involves replicating the conditions of the oral, gastric and intestinal phases of digestion under controlled pH levels, enzyme activities and digestion times. Although SGID was initially designed to study food digestion, it has become a valuable tool for generating food-derived peptides *in vitro*. Standardized protocols such as INFOGEST increase reproducibility (86, 87). The oral phase is typically excluded from peptide-focused studies (49).

In quinoa, pepsin alone generally results in partial hydrolysis, whereas sequential pepsin–pancreatin digestion promotes further breakdown and a marked shift toward low-molecular-weight peptides (88). Under SGID conditions, reported degrees of hydrolysis reach approximately 21.9%, depending on substrate and digestion parameters (30). Size-distribution analyses consistently show extensive generation of small peptides, with quinoa digests containing predominantly peptides <2000 Da (98.4%) and, in some cases, more than 40% of peptides < 200 Da (49). Matrix and processing effects further modulate digestion outcomes; for example, whole-food systems such as quinoa beverages yielded ~93.8% of peptides <1,000 Da after SGID (89), while germinated quinoa protein produced similarly high proportions of peptides <1,000 Da following sequential digestion (67). Pretreatments such as heating or protein fractionation have also been shown to influence hydrolysis efficiency, increasing globulin digestibility under pepsin–pancreatin digestion (59).

Physiological differences in digestion have also been explored using age-adapted SGID models. Comparative studies indicate that adult digestion models generate a higher proportion of very small peptides, with more than 60% of peptides below 1900 Da, whereas elderly models retain a greater proportion of larger peptides, reflecting age-related reductions in digestive efficiency (78).

Across other Andean grains, SGID outcomes remain strongly substrate-dependent. Using INFOGEST conditions, protein digestibility increased to 79.19% in amaranth concentrates and to 71.22% in cañihua concentrates (90), suggesting that cañihua is less digestible under comparable conditions. In amaranth, combining alcalase hydrolysis with SGID increased digestibility compared to alcalase alone, thus supporting the effectiveness of sequential enzymatic strategies in enhancing peptide generation (62). In lupin, the use of sequential digestion strategies substantially increased the release of peptides. The incorporation of alcalase following pepsin–pancreatin digestion increased DH from 17.8 to 46.12%, highlighting the potential of multi-enzyme configurations to overcome substrate resistance (91).

**Figure 2:**
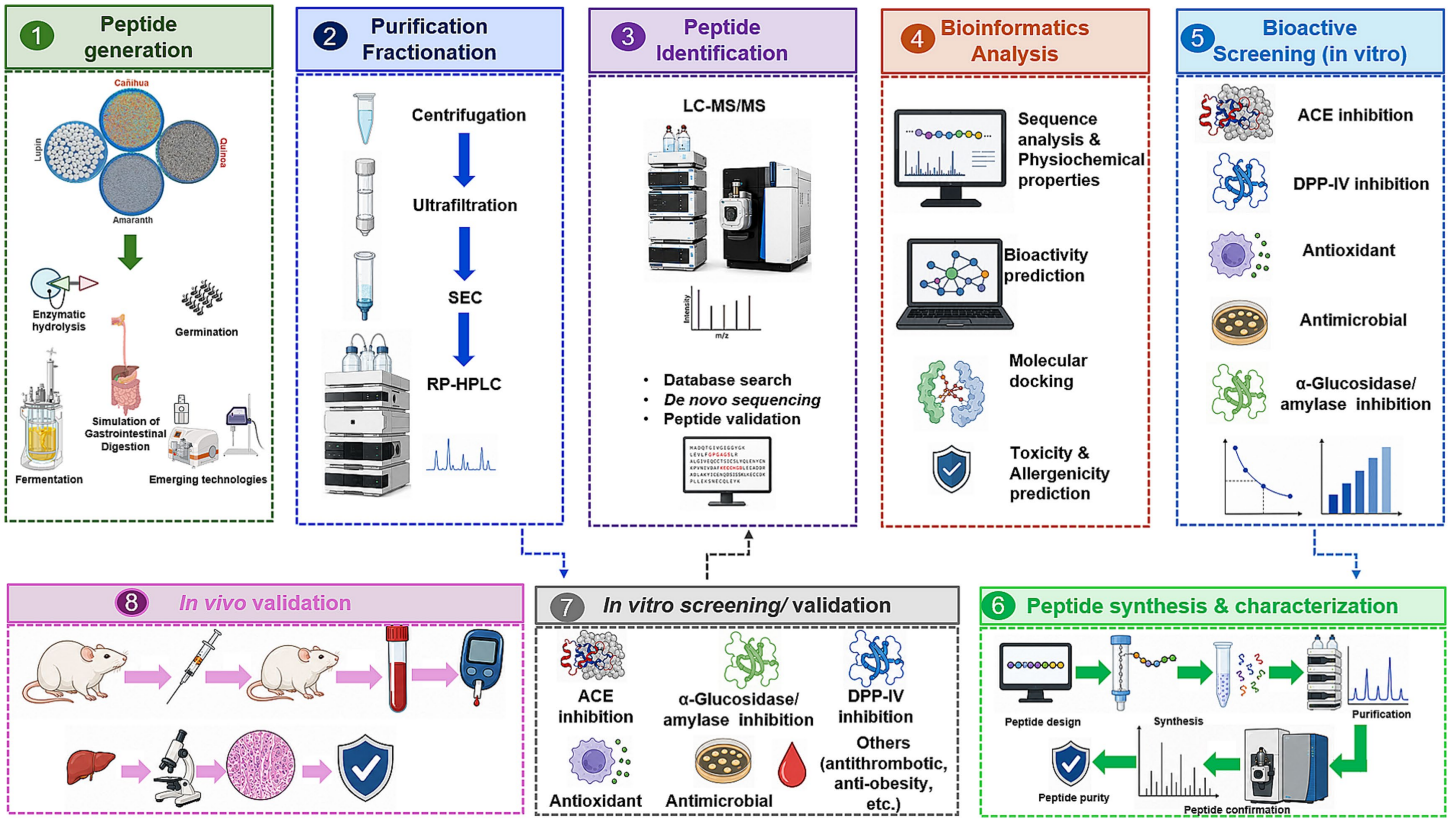
Workflow for the generation, identification, and biological validation of bioactive peptides from Andean grains.

Finally, amino acid profiling after SGID consistently reveals enrichment of hydrophobic residues (e.g., Tyrosine, Methionine, Valine, Isoleucine, Phenylalanine, Proline, Leucine) in digests from quinoa, amaranth, and lupin. This pattern could reflect digestion-driven peptide restructuring rather than uniform increases in antioxidant activity (11, 67, 90). As Di Stasio et al. (92) have highlighted, SGID models do not fully reproduce the complexity of the human gastrointestinal tract. Consequently, the biological relevance of the results should be interpreted with caution (Figure 2).

## 4 Separation, purification and identification of bioactive peptides from Andean grains

### 4.1 Separation and purification of bioactive peptide

Protein hydrolysates are complex mixtures of peptides that differ in size, charge and biological bioactivity, which require the use of selective separation and purification strategies. Ultrafiltration is the most commonly applied approach, employing membranes with cut-offs ranking from 1 to 50 kDa. For Andean grains, membranes with cut-offs of 1, 3, 5, 10, 30 and 50 kDa have been used, either individually or in sequential configurations, to obtain peptide fractions with enhanced bioactivity (30, 33, 38, 52, 61, 65, 77, 91). Sequential ultrafiltration has been proven particularly effective; for example, fractionation of cañihua protein hydrolysates using successive membrane cut-offs resulted in peptide reactions with improved biological activity (19). Despite its operational simplicity and relative low cost, ultrafiltration presents limitations, including low selectivity, peptide loss and membrane fouling (93–95).

To improve selectivity, electrodialysis with ultrafiltration membranes (EDUF) uses a combination of charge- and size-based separation. In quinoa, EDUF using 20 kDa membranes was employed to separate peptides into anionic (1.18%), cationic (3.01%), and neutral or non-migrating fractions (>20 kDa, ~95%) (31). Each of these fractions exhibited distinct bioactivities; for example, the anionic fraction improved glucose uptake *in vitro*, while neutral fractions showed antihypertensive activity *in vivo*; however, peptide recovery yields were lower than those achieved using ultrafiltration alone (31, 32).

Chromatographic methods are widely applied when higher-resolution purification is required. Size-exclusion chromatography (SEC) separates peptides according to their apparent molecular size, and has been used for fractions within the 100–5,000 Da range (17, 19, 63, 96). Reverse-phase high-performance liquid chromatography (RP-HPLC), most commonly using C18 columns and acetonitrile–water gradients, offers superior resolution based on peptide hydrophobicity. It has been extensively applied to Andean grains (25, 51, 58, 61, 72). For example, to identify antimicrobial peptides (36).

Finally, Ion-exchange chromatography (IEC) provides high-resolution, charge-based separation. This method has been successfully used to isolate anticancer proteins from quinoa seeds using DEAE cellulose (97), characterize Chenopodin isoforms (98), and purify antimicrobial peptides from cañihua hydrolysates using DEAE-Sephadex (17). Overall, combining membrane-based and chromatographic approaches may improves the efficiency of purification and enables more robust characterisation of bioactive peptides. However, these strategies tend to be more labor-intensive and less suitable for large-scale applications.

### 4.2 Peptide identification

Peptide identification is a critical, high-resolution step in the discovery of bioactive peptides and is performed after purification. The most widely used technique for this is liquid chromatography-mass spectrometry (LC–MS/MS) (99). This technique enables sequencing through peptide fragmentation and comparison against curated databases, such as UniProt (33, 36, 100, 101) or NCBI (97, 102).

Mass spectrometry is highly regarded for its ability to accurately determine molecular mass, amino acid composition and peptide sequences. Popular platforms include LC–MS/MS, UPLC–MS/MS, and nanoLC–MS/MS, which enable the sensitive separation, detection, and characterization of peptides (19, 30, 35, 66, 77, 89). Advanced systems such as the Orbitrap ELITE (103), Q Exactive Orbitrap (19), and Fusion Lumos (76) (Thermo Fisher Scientific), and the timsTOF Pro (32) (Bruker Daltonics) offer high resolution, making them particularly suitable for comprehensive proteomic studies.

In contrast, triple quadrupole instruments such as the Qtrap 6,500 + (SCIEX) are mainly employed for targeted peptide validation due to their high sensitivity. This approach has been demonstrated in the analysis of DPP-IV inhibitory peptides in quinoa malt, while Capraro et al. (29) used the same system to characterize chenopodin isoforms. However, limited untargeted discovery capability limited their application (72). Other systems, including the TripleTOF 5600 (SCIEX) and the Agilent 6,520 (Agilent Technologies), have accurately and efficiently identified more than 100 peptides in quinoa (93, 104).

Ion trap spectrometers, such as the LCQ Deca XP Max (Thermo Fisher Scientific) and the Esquire 3,000 (Bruker Daltonics), offer an affordable option with reasonable sensitivity, despite having lower resolution (15, 77). As a complementary approach, MALDI–TOF enables the rapid confirmation of peptide masses without the need for chromatographic separation, making it a useful tool for screening and fingerprinting. Although its resolution is lower than that of Orbitrap systems, using LIFT mode enables additional fragmentation patterns (31, 84).

Overall, although Orbitrap platforms (e.g., Q Exactive) remain widely used platforms for high-resolution peptide discovery, Q-TOF and MALDI–TOF/TOF offer more accessible options for validation and targeted analysis. The choice of platform ultimately depends on the objectives of the study and the balance between resolution, sensitivity and cost.

## 5 *In vitro* studies on bioactive peptides from Andean grains

### 5.1 Bioactivity of peptides obtained through enzymatic hydrolysis

This section focuses solely on peptides obtained through enzymatic hydrolysis. Various bioactive properties of Andean grain peptides were determined *in vitro* and are summarized in Table 2, whereas an extended compilation of studies is provided in Supplementary Table S2. Also, bioactivity data reported for peptides derived from Andean grains exhibit substantial heterogeneity in terms of experimental models, analytical assays, reference standards, and expression units. Depending on the bioactivity evaluated, results are reported as IC₅₀ values, percentage inhibition, Trolox equivalents, enzyme activity units, or physiological biomarkers. Due to this methodological diversity, complete standardization of units across studies was not feasible. Therefore, bioactivity results are presented using the original units reported by each study to preserve methodological accuracy and transparency.

a Antioxidant Activity of peptides released from Andean Grains

**Table 2:** *In vitro* studies of bioactive peptides from Andean grains generated by enzymatic hydrolysis.

Bioactive activity	Treatment	Source / fraction	Model	Assessment method	Main result	Source
Amaranth						
Antimicrobial	Hydrolysis (Protease, chymotrypsin, bromelain)	Protein Hydrolysate	Bacteria *(Staphylococcus aureus, Salmonella typhimurium, Escherichia coli)*	Well diffusion assay	*S. aureus*: Chymotrypsin (6 h) – 22.5 mm. *E. coli*: Chymotrypsin (6 h) – 18.5 mm. *E. aerogenes*: Protease (4 h) – 20.0 mm. *S. typhimurium*: Protease (2 h) – 16.5 mm.	(37)
Anti-hemolytic	Hydrolysis (Bromelain)	Protein Isolate	Cells (Human Erythrocytes)	Hemolysis inhibition assay	Hydrolysate at 6 h: IC₅₀ = 0.69 μg/mL; erythrocyte protection observed	(37)
Antioxidant	Hydrolysis (Bioprotease LA-660)	Protein Isolate	-	DPPH radical scavenging assay	Hydrolysate with 79.91 % inhibition and similar to the positive control	(14)
Cañihua						
Antihypertensive	Sequential Hydrolysis (Neutrase - Alcalase)	Protein Concentrate	-	Enzyme inhibition assay (ACE)	Hydrolysate at 180 min: IC₅₀ = 0.12 mg/mL; fraction <3,000 Da: IC₅₀ = 0.05 mg/mL	(19)
Antimicrobial	Hydrolysis (Alcalase)	Glutelin Fraction	Microorganisms (*E. coli*)	Bacterial growth Inhibition assay	Glutelin hydrolysate (4 h, 1:10) inhibited 95% of *E. coli* growth.	(17)
Lupin						
Antihypertensive	Sequential Hydrolysis (Alcalase - Neutrase)	Fraction <3,000 Da	-	Enzyme inhibition assay (ACE)	IC₅₀ = 0.06 mg/mL; peptide YSGWLGL (IC₅₀ = 0.14 μmol/L)	(103)
Antidiabetic	Sequential Hydrolysis (Alcalase - Neutrase) + Ultrafiltration + SEC	Fraction <3,000 Da	-	Enzyme inhibition assay (DPP-IV)	IC₅₀ = 0.32 mg/mL; peptide YSGWLGL identified (IC_50_ = 16.4 μmol/L)	(103)
Antioxidant	Hydrolysis (Alcalase vs. *Pseudomonas* sp.)	Protein Hydrolysate	-	ORAC assay	3.47 μmol TE/mg (Alcalase) > 1.03 μmol TE/mg (*Pseudomonas)*	(64)
Antioxidant	Sequential Hydrolysis (Alcalase - Neutrase)	Fraction <3,000 Da	-	ABTS radical scavenging assay	6.41 μmol TE/mg	(103)
Quinoa						
Antihypertensive	Hydrolysis (Neutrase) + Ultrafiltration + SEC	Peptide Fraction V-SEC (<3,000 Da)	-	Enzyme inhibition assay (ACE)	IC₅₀ = 39.1 μg/mL; 7.7-fold improvement vs. initial hydrolysate (IC₅₀ = 300.7 μg/mL)	(65)
Antihypertensive	High Hydrostatic Pressure (300 MPa) + Hydrolysis (Alcalase)	Protein Isolate	-	Enzyme inhibition assay (ACE)	86.49% at 0.5 mg/mL vs. 76% (control)	(51)
Antihypertensive	Fermentation (*P. ostreatus*) + Drying	Fermented Flour	-	Enzyme inhibition assay (ACE)	60–80%; increased after drying at 70 °C	(78)
Antidiabetic	Hydrolysis (Alcalase)	Protein Hydrolysate (<1,000 Da)	-	Enzyme inhibition assay (DPP-IV)	IC₅₀ = 1.23 mg/mL; peptides WLAFR and LLPFR identified	(33)
Antidiabetic	Hydrolysis (Alcalase) + EDUF fractionation	Neutral Fraction (QPH-EDUF)	-	Enzyme inhibition assay (DPP-IV)	IC₅₀ = 1.07 mg/mL	(32)
Antidiabetic	Malting (Germination)	Malted Quinoa	-	Enzyme inhibition assay (DPP-IV)	Malting (15 °C, 3 days) 45.02% inhibition vs. unmalted	(72)
Antidiabetic	Hydrolysis (Alcalase) + HHP	Protein hydrolysate (<3,000 Da)	-	Enzyme inhibition assay (DPP-IV)	Alcalase hydrolysate (<3,000 Da) at 300 MPa: DPP-IV 56.27% (0.1 mg/mL) inhibition; 86.49% (0.5 mg/mL) and IC_50_ = 0.07 mg/mL	(51)
Antihypertensive	HPH + Hydrolysis (Alcalase)	Protein hydrolysate (<3,000 Da)	-	Enzyme inhibition assay (ACE)	91.5–93.2% at 0.5 mg/mL; 50 MPa showed highest inhibition 92.6%; IC₅₀ = 0.04 mg/mL)	(82)
Antidiabetic	Hydrolysis (Alcalase) + EDUF fractionation	Anionic Peptide Fraction	Cells (L6 skeletal muscle)	Glucose uptake assay	Glucose uptake increased by 17.9% (1 μg/mL, insulin-stimulated) vs. control	(31)
Anti-inflammatory	Purification (Ion Exchange)	*Chenopodin* isoforms (LcC, HcC)	Cells (Caco-2)	RT-qPCR (IL-8 expression, NF-κB pathway)	Isoform LcC reduced IL-8 expression by 53% and inhibited NF-kB activation (~30%) HcC reduced IL-8 by 38%.	(98)
Anti-inflammatory	Purification (UPLC + Ion Exchange)	Lunasin	Cells (RAW264.7)	NO production assay (Griess)	NO inhibition: 44.8%; TNF-*α* (39.8%) and IL-6 (33.5%)	(73)
Antimicrobial	Fermentation (Mixed bacteria)	Peptide AGAAPE (<1,000 Da)	Bacteria (*E. coli*, *S. aureus*)	MIC assay (broth microdilution)	MIC = 5 mg/mL; membrane disruption observed	(36)
Antimicrobial	Hydrolysis (Pepsin + Alcalase)	Protein Hydrolysate	Bacteria (*S. pyogenes*, *E. coli*)	Agar well diffusion assay	Maximum inhibition zones of 11.88 mm (*S. pyogenes*) and 12.49 mm (*E. coli*) were achieved at 0.80 mg/mL.	(121)
Antimicrobial	Hydrolysis (Protease, chymotrypsin, bromelain)	Protein Hydrolysate	Bacteria *(Staphylococcus aureus, Salmonella typhimurium, Escherichia coli)*	Well diffusion assay	*S. aureus*: Bromelain (6 h) – 19.5 mm *S. typhimurium*: Bromelain (4 h) – 20.0 mm *E. coli*: Protease (4 h) – 19.0 mm *E. aerogenes*: Protease (2 h) – 20.0 mm	(37)
Antioxidant	Hydrolysis (Alcalase)	Protein Hydrolysate (<1,000 Da)	-	DPPH radical scavenging assay/ABTS radical scavenging assay	63.96% (DPPH); 88.43% (ABTS) at 2.5 mg/ml; IC₅₀ = 3.08 mg/mL (DPPH); IC₅₀ = 0.12 mg/mL (ABTS)	(33)

EDUF, Electrodialysis with Ultrafiltration Membranes; HHP, High Hydrostatic Pressure; SEC, Size Exclusion Chromatography; MIC, Minimum Inhibitory Concentration; IC50, Half-Maximal Inhibitory Concentration; NO, Nitric oxide; HPH, High-Pressure Homogenization.

Research on quinoa proteins has repeatedly identified alcalase as one of the most effective enzymes for generating antioxidant peptides. Hydrolysates produced using alcalase have been found to have IC₅₀ values of 0.57 mg/mL (ABTS) and 1.95 mg/mL (hydroxyl radicals), which is better than the results obtained using flavourzyme, papain and neutral protease (49). Similarly, Gan et al. (33) reported an IC₅₀ value of 0.28 mg/mL and 90% ABTS inhibition approaching the activity of ascorbic acid. The enhanced performance of alcalase has been attributed to its preferential release of low-molecular-weight, hydrophobic peptides enriched in tyrosine, methionine, valine, isoleucine, glycine, phenylalanine, proline and leucine, which accounted for approximately 27% of the identified sequences (49). However, antioxidant performance depends not only on enzyme selection, but also on enzyme specificity and peptide cleavage patterns. Chymotrypsin-derived hydrolysates have demonstrated comparable or even higher ABTS and DPPH scavenging activities than alcalase under specific conditions (37), while sequential hydrolysis enhanced antioxidant activity compared with single-enzyme systems (13, 91). Likewise, Y. Wang et al. (55), demonstrated that dual-enzyme systems using papain and alcalase generated hydrolysates with greater antioxidant activity that either enzyme alone. In contrast, Gao et al. (54) reported that although alcalase achieved the highest HD among six proteases, pepsin hydrolysates exhibited superior antioxidant capacity owing to the preferential release of peptides enriched in aromatic and basic amino acid residues. These findings indicate that antioxidant activity is governed more by enzyme specificity and peptide composition than by hydrolytic efficiency alone.

Across quinoa and other Andean grains, fractionation studies consistently demonstrate higher activity in low-molecular-weight fractions. Peptides <1,000 Da showed greater antioxidant capacity than those < 3,000 Da (33, 65), whereas peptides <3,000 Da outperformed fractions <10 kDa, with ABTS scavenging exceeding 75% in some cases (7). Similar trends were observed in cañihua and lupin, where <3,000 Da fractions exhibited superior activity (19, 91). The enhanced activity of smaller peptides may be associated with their greater mobility and accessibility to reactive species. However, molecular size alone does not explain antioxidant behavior, which also depends on peptide sequence and amino acid composition. For instance, Chirinos et al. (103) reported that the < 3,000 Da lupin peptide fraction did not exhibit greater antioxidant activity than the entire protein hydrolysate, suggesting that the combined effects of peptides may enhance overall antioxidant capacity. Similarly, lunasin efficiently scavenged ABTS radicals but showed limited DPPH activity (73), highlighting the importance of peptide sequence and assay-specific reaction mechanisms. Furthermore, the relationship between peptide size, degree of hydrolysis, and antioxidant activity in quinoa is not strictly linear. Rueda et al. (85) demonstrated that maximum radical-scavenging activity was achieved at a low degree of hydrolysis (2-5%), whereas prolonged hydrolysis reduced antioxidant capacity by degrading bioactive peptide sequences. Nevertheless, Wang et al. (55) confirmed that isolated peptides within 1-3 kDa and <1 kDa ranges consistently exhibit superior electron transfer capacity owing to reduced steric hindrance (105).

Processing strategies can further modulate antioxidant activity by modifying protein structure before or during hydrolysis. For example, high hydrostatic pressure combined with alcalase hydrolysis was found to significantly increase antioxidant values (51), while ultrasound pretreatment (400 W) was shown to enhance ABTS, DPPH and ORAC values by 65–75% (83). Fermentation with *Lactobacillus plantarum* produced short peptides (5–9 amino acids) with antioxidant activity that was superior to that of synthetic antioxidants such as BHT (77). Similarly, fermentation of quinoa with *Rhizopus oligosporus* improved antioxidant activity (80). These findings suggest that structural modification of the protein matrix facilitates peptide release; however, the extent of this effect depends on processing intensity. Mild pre-treatments, such as germination and extrusion, increase protein susceptibility to enzymatic hydrolysis and maximize antioxidant activity at low degrees of hydrolysis (85), whereas severe thermal processing, such as cooking at 120 °C before dual-enzyme hydrolysis, reduced antioxidant capacity by promoting the degradation or oxidation of amino acid residues involved in radical scavenging (55). Furthermore, some processing technologies may simultaneously promote the release of phenolic compounds, which could also contribute to the antioxidant activity observed (51).

Although most studies assessed antioxidant activity using chemical assays such as ABTS, DPPH, ORAC or hydroxyl radical methods (37, 49), these methods do not necessarily predict physiological antioxidant effects *in vivo* and should therefore be interpreted with caution. Nevertheless, further animal studies and human clinical trials are required to confirm these findings. Xiao et al. (66) identified the quinoa derived peptide GPGGGGKGEMF, which scavenges radicals and activates the Keap1–Nrf2 pathway, thereby enhancing endogenous antioxidant defences. Similarly, synthetic peptides such as SW and IW exhibited moderate DPPH scavenging activity (32–35%) (106). However, some peptides reduced Caco-2 cell viability at high concentrations, while exerting cytoprotective effects under oxidative stress conditions. These findings emphasize that antioxidant functionality is context-dependent and influenced by dose, peptide sequence and evaluation model.

Overall, the antioxidant activity of peptides derived from Andean grains results from the combined influence of enzyme specificity, peptide molecular weight, amino acid composition, processing strategy, and the biological model used for evaluation. Rather than depending on a single structural or technological factor, antioxidant performance reflects the interaction among these variables.

b Antidiabetic Activity of peptides released from Andean Grains

The inhibition of dipeptidyl peptidase IV (DPP-IV), *α*-amylase and α-glucosidase is a key mechanism by which food-derived peptides can help to control blood sugar levels, by prolonging incretin activity and reducing the absorption of glucose after eating. In quinoa, intact proteins exhibit modest DPP-IV inhibition (IC₅₀ > 2–11.8 mg/mL) (24, 33, 107), whereas enzymatic hydrolysis significantly increases activity. Alcalase-derived hydrolysates exhibit IC₅₀ values of 1.28–1.32 mg/mL (32, 33), decreasing to 0.62 mg/mL following fractionation and lipid removal (32). Other proteases, including bromelain, chymotrypsin, trypsin, flavourzyme, papain and pronase E, produce hydrolysates with IC₅₀ values ranging from 0.72 to 4.70 mg/mL (33, 107). Notably, chymotrypsin yields highly potent fractions under specific conditions for inhibition of DPP-IV (107). These differences likely reflect enzyme-specific cleavage patterns that shape peptide profiles, rather than any inherent superiority of the enzymes involved (33). More recently, *in vitro* validation demonstrated that the peptide QDQHQKIR, containing a C-terminal arginine residue, preferentially inhibited *α*-amylase, whereas NIYQIS and YDDER showed greater specificity toward DPP-IV and *α*-glucosidase, respectively (108).

The molecular weight of peptides influences their inhibitory potency. The <1,000 Da fraction obtained via alcalase hydrolysis of quinoa was found to inhibit DPP-IV, *α*-amylase and α-glucosidase with IC₅₀ values of 1.23, 3.66 and 3.31 mg/mL, respectively (33). This fraction contained short peptides, including WLAFR and LLPFR (33). Similarly, Abbasi et al. (7) observed stronger α-glucosidase inhibition in trypsin-generated peptides <3,000 Da (44.79%) than in larger fractions. Despite exhibiting measurable activity, food-derived peptides remain less potent than pharmaceutical inhibitors such as Sitagliptin (DPP-IV) and Acarbose (*α*-amylase/α-glucosidase) (33), highlighting the difference between functional bioactivity and therapeutic efficacy.

Amino acid composition also could contribute to activity antidiabetic. Potent quinoa hydrolysates were enriched in hydrophobic (L, A, V, F) and aromatic residues, accounting for approximately 20.89 and 5.31% of total amino acids, respectively (33). Hydrolysis time is another critical factor, as prolonging the reaction reduced *α*-glucosidase inhibition in both trypsin and alcalase-derived fractions (7). Thermolysin hydrolysates (24 h) exhibited DPP-IV IC₅₀ values of 2.40 mg/mL, whereas hydrolysed quinoa globulin fractions reached 3.37 mg/mL (11). This highlights the influence of enzyme selection and protein source on activity.

Processing strategies that modify protein conformation can further diversify peptide generation. De Carvalho Oliveira et al. (82) found that high-pressure homogenization (50 MPa) followed by alcalase hydrolysis promoted the release of multifunctional peptides. Peptidomic analysis identified sequences such as SPGYYDGR, which has predicted α-glucosidase inhibitory potential. The tripeptide SPH, which is derived from quinoa globulin, exhibited stronger DPP-IV inhibition (IC₅₀ = 0.54 mg/mL) than crude hydrolysates (11). Germination at 15 °C for 3 days also enhanced DPP-IV inhibitory activity (72).

Beyond direct enzyme inhibition, emerging evidence suggests that Andean grain peptides may influence glucose metabolism via cellular mechanisms. For example, González-Muñoz et al. (31) demonstrated that the neutral fraction of quinoa hydrolysates increased basal glucose uptake in L6 muscle cells by 17.1%. In contrast, the anionic fraction was more effective under insulin stimulation, increasing uptake by 17.9%. This suggests that the charge of peptides can modulate metabolic pathways differently (31).

Similar trends were observed in lupin, where whole hydrolysates exhibited stronger DPP-IV inhibition than purified <3,000 Da fractions (103), suggesting potential synergistic effects within complex mixtures. Multifunctional peptides such as YSGWLGL further illustrate pleiotropic bioactivity (109).

Overall, the antidiabetic potential of Andean grain peptides depends on peptide structure, molecular weight distribution, amino acid composition, hydrolysis conditions and enzyme–peptide interactions. However, methodological heterogeneity and the limited number of *in vivo* and clinical studies currently restrict conclusions regarding their physiological relevance and therapeutic potential.

c Anti-inflammatory Activity of Peptides Released from Andean Grains

The inflammatory response is regulated by interconnected signaling pathways, including mitogen-activated protein kinases (MAPK), nuclear factor kappa B (NF-κB) and phosphatidylinositol-3-kinase/protein kinase B (PI3K/Akt). These pathways coordinate the production of cytokines and the cellular response to oxidative stress (110–112). NF-κB is particularly important in regulating the transcription of pro-inflammatory mediators, including nitric oxide (via inducible nitric oxide synthase), tumor necrosis factor-*α* (TNF-α), interleukin-6 (IL-6) and interleukin-8 (IL-8). These molecules are widely recognized as key amplifiers of inflammatory cascades and are frequently used as biomarkers in *in vitro* models of inflammation (113).

*In vitro* studies have evaluated the anti-inflammatory potential of quinoa-derived peptides in this context. Lunasin, a naturally occurring quinoa peptide, was found to have no cytotoxic effects on RAW 264.7 macrophages at concentrations of 0.25–0.40 mg/mL, while significantly reducing the production of nitric oxide (NO), TNF-α and IL-6 under lipopolysaccharide (LPS) stimulation (73). The influence of enzymatic hydrolysis on the anti-inflammatory activity of quinoa proteins remains unclear. While Shi et al. (60) found no significant differences between intact and hydrolyzed quinoa proteins, Tocmo et al. (114) reported that hydrolysis using flavourzyme and papain significantly increased the inhibition of pro-inflammatory mediators compared with intact proteins. These contrasting findings suggest that the anti-inflammatory potential of quinoa proteins may depend on the hydrolysis method employed and the resulting peptide profile.

Specific storage protein fractions in quinoa, beyond total hydrolysates, have shown immunomodulatory effects. Capraro et al. (98) demonstrated that chenopodin isoforms significantly modulate inflammatory signaling in IL-1β-stimulated Caco-2 intestinal epithelial cells. The LcC (low-charge chenopodin) and HcC (high-charge chenopodin) isoforms were found to inhibit NF-κB activation by 30–45% and reduce IL-8 expression by 38–53%, suggesting the potential to mitigate epithelial inflammatory responses at the intestinal level (98).

Although recent studies have improved understanding of the molecular mechanisms underlying the anti-inflammatory activity of quinoa peptides, the available evidence remains limited as only a small number of studies have evaluated this bioactivity. Further *in vivo* and clinical studies are therefore required to clarify the physiological relevance and therapeutic potential of quinoa peptides.

d Antihypertensive Activity of Peptides Released from Andean Grains

Hypertension is primarily mediated by the renin–angiotensin system, in which angiotensin-converting enzyme and renin regulate blood pressure through the generation of vasoconstrictor peptides and the degradation of vasodilators (30). Food-derived peptides can inhibit these enzymes, providing a safer, more natural alternative to synthetic drugs such as captopril (115).

Native quinoa proteins exhibit moderate ACE inhibitory activity, which increases after enzymatic hydrolysis. Alcalase-treated quinoa protein isolate showed approximately 76% ACE inhibition (51). Suleman et al. (115) found that thermolysin-derived quinoa hydrolysates exhibited an IC₅₀ of 0.20 mg/mL of ACE (115), and chymotrypsin hydrolysates showed a similar IC₅₀ of 0.22 mg/mL (107). Neutrase hydrolysis generated the strongest ACE inhibition, with an IC₅₀ of 0.08 mg/mL (13). However, these values remain below those of captopril, indicating functional rather than pharmacological potency. Recent evidence also indicates that the degree of hydrolysis is an important determinant of ACE inhibitory activity. Rueda et al. (85) demonstrated that germination followed by alcalase hydrolysis achieved maximum ACE inhibition at 20% DH, highlighting that optimized hydrolysis conditions, rather than extensive protein degradation alone, determine the generation of potent ACE inhibitory peptides.

Peptide size also influences antihypertensive activity. In quinoa, fractions <3,000 Da obtained after neutrase hydrolysis exhibited a 7.7-fold increase in ACE inhibition compared to whole hydrolysates (65). Processing strategies further enhanced this activity. High hydrostatic pressure combined with alcalase increased ACE inhibition to 86.49% (51). High-pressure homogenization further improved ACE inhibition to 92.6%, corresponding to an IC₅₀ of 0.04 mg/mL (51). These technologies probably enhance peptide release by improving protein accessibility during hydrolysis.

Fermentation with *Lactobacillus casei* produced peptides such as IFRPFAPEL, which had an IC₅₀ of ACE value of 0.22 mg/mL (76). In contrast, *Pleurotus ostreatus* fermentation resulted in 60–80% ACE inhibition (78). Short peptides such as IW and PW exhibited 51 and 37% inhibition at 1 mg/mL (106).

In cañihua, alcalase–neutrase hydrolysates exhibited an ACE IC₅₀ value of 0.32 mg/mL for ACE inhibition. Sequential hydrolysis reduced this value to 0.12 mg/mL and the IC₅₀ of purified low-molecular-weight fractions was 0.05 mg/mL (19). In lupin, alcalase–neutrase hydrolysis yielded an ACE inhibitory IC₅₀ value of 0.10 mg/mL (103), whereas fractions <3,000 Da showed improved ACE inhibition with an IC₅₀ of 0.06 mg/mL. The peptide YSGWLGL exhibited micromolar potency in the ACE inhibition assay (103).

Overall, these studies show that *in vitro* ACE inhibitory activity is mainly determined by enzyme specificity, peptide size distribution, degree of hydrolysis and processing strategy. Sequential hydrolysis and peptide fractionation appear to enhance peptide bioactivity across different Andean grains. However, methodological heterogeneity limits direct comparisons between studies.

e Anti-haemolytic Activity of Peptides Released from Andean Grains

Anti-hemolytic activity reflects the ability of bioactive peptides to protect erythrocyte membranes against oxidative or enzymatic damage, and is commonly used as an indicator of membrane-stabilization and antioxidant potential (116, 117). The available evidence in Andean grains remains limited. Mudgil et al. (37) evaluated intact proteins and hydrolysates generated using bromelain, chymotrypsin and a neutral protease in an *in vitro* hemolysis model in quinoa and amaranth. The intact quinoa protein exhibited stronger protective activity (IC₅₀ = 0.0098 mg/mL) than the intact amaranth protein (IC₅₀ = 0.0174 mg/mL) (37).

Enzymatic hydrolysis may enhance anti-hemolytic capacity. The lowest IC₅₀ value (0.00069 mg/mL) was observed in amaranth hydrolysates obtained after 6 h of bromelain treatment, followed by quinoa hydrolysates produced with chymotrypsin (IC₅₀ = 0.00083 mg/mL) and protease (IC₅₀ = 0.00098 mg/mL) (37). These findings suggest that enzymatic hydrolysis promotes the release of peptides that can enhance erythrocyte membrane protection under the reported experimental conditions. However, the influence of peptide size, amino acid composition or structure–activity relationships on anti-hemolytic activity remains unclear due to insufficient evidence. Furthermore, methodological heterogeneity hinders direct comparisons between studies and prevents the elucidation of the molecular mechanisms underlying erythrocyte membrane protection.

f Antimicrobial Activity of peptides released from Andean Grains

Antimicrobial peptides (AMPs) derived from food proteins are increasingly recognized as a promising natural alternative to conventional antibiotics. Native plant proteins often exhibit little or no antimicrobial activity, but enzymatic hydrolysis and microbial fermentations can markedly enhance their functionality by releasing bioactive peptide sequences (118–120). Native quinoa and amaranth proteins exhibited limited antimicrobial activity. For example, Mudgil et al. (37) reported weak activity against *Salmonella typhimurium* by intact protein isolates. The available evidence suggests that enzymatic hydrolysis may enhance antimicrobial activity in a strain- and enzyme-dependent manner. In quinoa and amaranth, chymotrypsin-derived amaranth peptides exhibited the strongest inhibition against *S. aureus*, whereas quinoa hydrolysates produced using bromelain and protease were the most effective against *S. typhimurium* and *E. coli*, respectively. *E. aerogenes* inhibition was achieved by quinoa and amaranth hydrolysates produced using protease (37), demonstrating the combined impact of substrate and enzyme specificity. In cañihua, alcalase-derived hydrolysates inhibited *E. coli, S. aureus,* and *C. albicans*, while native proteins were inactive (17). A purified 7S globulin fraction exhibited moderate antimicrobial inhibition, suggesting that peptide charge distribution may influence membrane interactions in microorganism (17). Likewise, optimization of quinoa protein hydrolysis improved antimicrobial performance, reaching levels comparable to gentamicin under specific *in vitro* conditions against *S. pyogenes* and *E. coli*. (121). Recently, Wang et al. (55) reported that antibacterial activity was further enhanced by sequential hydrolysis with papain and alcalase, generating low-molecular-weight peptides (1–3 kDa) with improved membrane-disrupting capacity against *E. coli* and *S. aureus*. In contrast, high thermal pre-treatment reduced antimicrobial activity, emphasizing the importance of preserving peptide integrity during processing.

Fermentation represents an additional strategy for releasing antimicrobial peptides. Zhu et al. (36) identified the peptide AGAAPE with molecular weight < 1,000 Da in fermented quinoa, with a minimum inhibitory concentration (MIC) of 5 mg/mL against both *E. coli* and *S. aureus*. This antimicrobial action of AGAAPE was attributed to the membrane destabilization through hydrophobic interactions and hydrogen bonding, which is consistent with the presence of amphipathic structural features (36).

Overall, the available evidence suggests that enzymatic hydrolysis and fermentation may increase the antimicrobial effectiveness of peptides derived from Andean grains. However, differences in peptide composition, target microorganisms and antimicrobial assays currently prevent direct comparisons between studies, highlighting the need for standardized evaluation protocols.

### 5.2 Bioactivity of peptides obtained through simulation of gastrointestinal digestion

The *in vitro* bioactivities reported for peptides derived from Andean grains following simulated gastrointestinal digestion are summarized in Table 3, whereas an extended compilation of studies is provided in Supplementary Table S3.

a Antioxidant activity after digestion.

**Table 3:** *In vitro* studies of bioactive peptides obtained through SGID.

Treatment	Bioactive activity	Andean grain	Source / fraction	Model	Assessment method	Main result of bioactivity	Source
SGID (pepsin-pancreatin)	Anticancer	Amaranth	Gastric Digest (120 min)	Cells (Caco-2)	MTT Assay (IC_50_)	80.15% inhibition at 4 mg/mL	(15)
SGID (INFOGEST) + Ultrafiltration (>5,000 Da)	Cellular antioxidant	Amaranth	Intestinal digest (>5,000 Da)	Cells (HepG2)	Cellular Antioxidant Activity (CAA) assay	81.06% cellular antioxidant activity	(90)
SGID (pepsin-pancreatin)	Multifunctional	Amaranth	Intestinal Digest (60 min)	–	Enzyme inhibition assay (DPP-IV)	IC_50_ = 0.32 mg/mL	(15)
SGID (pepsin-pancreatin) + pre-hydrolyzed proteins (Neutrase)	Antioxidant	Amaranth	Digested hydrolysates	-	ABTS radical cation scavenging assay	No significant changes after digestion; ~ 1.71 μmol TE/mg vs. before 1.67 μmol TE/mg	(13)
Malting + SGID	Antidiabetic	Quinoa	Malted Digest	–	Enzyme inhibition assay (DPP-IV)	41.86% inhibition after malting	(72)
Germination + SGID	Antidiabetic	Quinoa	Germinated Digest	–	α-Glucosidase Inhibition	Germination (24 h) + digestion improved IC_50_ = 0.07 mg/mL vs. control IC_50_ = 0.24 mg/mL	(69)
Germination + SGID + Ultrafiltration (<1,000 Da)	Antidiabetic	Quinoa	Fraction <1,000 Da	Cells (Caco-2)	Enzyme inhibition assay (DPP-IV)	IC₅₀ = 2.20 mg/mL; activity confirmed *in situ*	(61)
Fermentation + SGID	Antihypertensive	Quinoa	Fermented Flour Digest	–	Enzyme inhibition assay (ACE)	Digestion increased ACE inhibition in fermented samples (30-80%) Fermentation + hot air-drying recovered ACE inhibition up to 60-80% due to melanoidins. Older adult GI conditions reduced ACE inhibition by up to 30% compared to the standard model	(78)
SGID (pepsin-pancreatin)	Antioxidant	Quinoa	Intestinal digest (120 min)	–	ORAC Assay	2.39 μmol TE/mg vs. 0.42 in intact protein	(25)
Heat treatment + SGID (pepsin-pancreatin)	Antihypertensive	Quinoa	Isolated globulin fraction		Enzyme inhibition assay (ACE)	100% inhibition after intestinal phase (pancreatin) and 5% (pepsin)	(59)
Enzymatic hydrolysis (Neutrase, Alcalase) followed by SGID (pepsin-pancreatin)	Antihypertensive	Quinoa; Amaranth	Quinoa (QPH) and Amaranth (APH) protein hydrolysates	*-*	Enzyme inhibition assay (ACE)	SGID did not alter the ACE-I inhibitory activity of the hydrolysates; IC₅₀ = 0.07 mg/mL (QPH) and 0.31 mg/mL (APH); activity retained after digestion	(13)

SGID, Simulated Gastrointestinal Digestion; ACE, Angiotensin-Converting Enzyme; DPPH, 2,2-diphenyl-1-picrylhydrazyl; ABTS, 2,2′-azinobis(3-ethylbenzothiazoline-6-sulfonic acid); GI, Gastrointestinal; ORAC, Oxygen Radical Absorbance Capacity; SOD, Superoxide Dismutase; CAT, Catalase; HepG2, Human Liver Cancer Cell Line; QPH, Quinoa Protein Hydrolysate; APH, Amaranth Protein Hydrolysate.

SGID influences the antioxidant capacity of Andean grain proteins by encouraging the release of peptides and altering their structure (25, 62, 90, 91). Digestion may expose reactive residues and generate low-molecular-weight peptides with enhanced radical-scavenging capacity rather than simply promoting protein degradation (49, 122).

In quinoa protein concentrates, for example, antioxidant activity increased from 0.42 μmol TE/mg (native protein) to 1.03 μmol TE/mg after gastric digestion, reaching 2.22–2.39 μmol TE/mg following the intestinal phase (25). Fractions of less than 5,000 Da exhibited the highest ORAC values (up to 2.72 μmol TE/mg), suggesting that reducing the molecular size may enhance the accessibility of functional groups. Similar trends have been reported in amaranth, cañihua and tarwi, where ultrafiltered fractions (<3,000–5,000 Da) exhibited stronger antioxidant and metal-chelating activity than crude hydrolysates (90, 91). SGID of quinoa has also been shown to produce predominantly small fragments (less than 2000 or 1,000 Da), which collectively maintain high DPPH, ABTS and FRAP activities (49, 67), the composition of peptides further influences post-digestive antioxidant performance. Aromatic residues (W, Y), sulfur-containing residues (M, C) and hydrophobic residues (L, V, I) have been associated with enhanced radical-scavenging efficiency (49). Sequential pepsin–pancreatin digestion often yields stronger responses than single-enzyme treatments, with substantial increases reported under specific conditions (59).

Nevertheless, enhancement after digestion is not universal. Some studies have reported only modest changes following SGID (13), which suggests that conformational masking, redistribution of peptides, or the stability of pre-existing antioxidants may influence outcomes (49). Acidic gastric conditions may transiently conceal reactive sites, which are subsequently re-exposed during intestinal hydrolysis.

Cellular models extend these observations beyond chemical assays. Intestinal digestion of amaranth and cañihua proteins enhanced the intracellular antioxidant response in both the HepG2 and Caco-2 systems without evident cytotoxicity (90). This supports the physiological relevance of digestion-derived peptides.

Overall, antioxidant bioactivity after digestion depends on the interplay between peptide size, amino acid composition, enzymatic specificity and digestion protocol. However, variability in simulated digestion models and analytical assays limits direct cross-study comparison (25, 91).

b Antidiabetic activity after digestion.

Digestion-derived peptides from Andean grains such as quinoa and amaranth have been increasingly associated with antidiabetic potential, particularly through the inhibition of DPP-IV, *α*-amylase, and α-glucosidase (123). Native protein concentrates generally exhibited limited inhibitory activity, whereas SGID significantly enhances bioactivity, in low-molecular-weight fractions <5,000 Da (88).

In quinoa, gastric digestion with pepsin releases DPP-IV-inhibitory peptides (IC₅₀ = 2.52 mg/mL). Subsequent pancreatin digest markedly improves DPP-IV inhibition (IC_50_ = 0.23–0.25 mg/mL) and additionally induces *α*-amylase and α-glucosidase inhibitory activities (IC_50_ = 0.19 and 1.81 mg/mL, respectively). Fractions < 5,000 Da consistently exhibit stronger inhibition across the three enzymes. Sequences such as IQAEGGLT, DKDYPK and GEHGSDGNV derived from the 11S B storage globulin have been identified as key contributors (88). For instance, IQAEGGLT acts as potent α-glucosidase inhibitor due to its hydrophobic residues, though it lacks the aromatic residues required for α-amylase inhibition (88).

Germination of quinoa for short time (2 h) followed by pepsin-trypsin digestion generated peptides with potent DPP-IV inhibitory activity. Particularly, fractions <1,000 Da exhibited remarkable activity in both *in vitro* (IC_50_ = 3.40 mg/mL) and Caco-2 (IC_50_ = 2.20 mg/mL) assays, revealing peptides sequences such as IPI and IPV (61). Increasing the germination time (24 h) boosted the *α*-glucosidase inhibitory activity to 81.1% at a concentration of 4 mg/mL (IC₅₀ = 0.07 mg/mL against a human homolog) (69). Systematic malting also breaks down storage globulins and albumins into oligopeptides that, upon SGID, release high concentrations of specific DPP-IV inhibitor such as APF and HI, and achieve a total DPP-IV inhibition of up of 41.86% (72).

Amaranth exhibits a similar digestion-dependent enhancement. Gastric digestion increases DPP-IV inhibition, which intensifies during gastroduodenal digestion (60-120 min), reaching IC₅₀ values of 0.32 and 0.28 mg/mL, respectively. Intestinal fractions < 5,000 Da display stronger DPP-IV inhibition (0.19 mg/mL), and peptides with low-molecular-weight (630–945 Da) have shown comparable potency (IC₅₀ = 0.18 mg/mL). For *α*-amylase inhibition, the IC₅₀ values were 0.84 mg/mL for the 120 min digest and 1.17 mg/mL for the 505–669 Da fraction. Also, identified sequences such as FLISCLL, SVFDEELS and DFIILE displayed dual antidiabetic activities (15).

Importantly, the apparent potency of α-glucosidase inhibitors may vary depending on enzyme source (rat intestinal versus microbial origin), which complicates direct cross-study comparisons (124). Overall, the available evidence suggests that SGID may improve the antidiabetic potential of quinoa and amaranth proteins, although the magnitude of inhibition depends on peptide size, amino acid composition, digestion protocol, and assay conditions.

c Anticancer Activity After Digestion.

Peptides released during SGID from quinoa and amaranth proteins have been associated with selective cytotoxic effects on tumor cell lines, highlighting their ability to modulate cell viability in colorectal cancer models (15).

In quinoa, testing on Caco-2 cells revealed that only the specific HPLC fraction F-3 exhibited significant activity, with an IC₅₀ of 2.56 mg/mL (25). In contrast, all fractions (F-1, F-2 and F-3) affected HT-29 and HCT-116 cells, although F-3 remained the most potent (IC₅₀ = 0.195 and 0.193 mg/mL, respectively). These differences suggest cell-line-dependent sensitivity and potential selectivity to colorectal cancer models. Similarly, amaranth digests reduced Caco-2 viability (IC₅₀ = 1.17 and 0.87 mg/mL for F-1 and F-2, respectively) (15).

The influence of molecular weight on anticancer activity remains less consistent. Some studies report that low-molecular-weight quinoa protein hydrolysates < 5,000 Da inhibited Caco-2 proliferation by approximately 53.9% at 8 g/L, suggesting enhanced antiproliferative potential of smaller peptides (35). However, other investigations evaluating SGID-derived fractions from quinoa and amaranth have observed lower anticancer activity in <5,000 Da fractions compared to larger peptides (15). Such discrepancies may not be attributable solely to molecular size but could also reflect differences in cultivar., environmental conditions, peptide composition, digestion protocol, or experimental design.

Overall, digestion-derived peptides from some Andean grains may show selective antiproliferative effects in colorectal models, although methodological variability limits cross-study comparability.

d Antihypertensive Activity after Digest.

ACE inhibitory activity following simulated gastrointestinal digestion depends on the sequence of the enzymes involved, the stability of the peptides, and the strategy used for fractionation. Although digestion can enhance the antihypertensive potential by releasing bioactive peptides, further hydrolysis can also reduce activity through peptide degradation.

In quinoa, SGID may markedly improve ACE inhibition through progressive protein breakdown. Pepsin alone results in limited ACE inhibition (approximately 5%), whereas subsequent pepsin–pancreatin digestion achieves complete ACE inhibition (59). This highlights the importance of sequential hydrolysis. Size fractionation further strengthens this effect, where fractions < 5,000 Da reduce IC₅₀ values to approximately 0.16 mg/mL, and further purification can lower IC₅₀ to ~0.05 mg/mL (93). Potent digestion-derived peptides, such as NWFPLPR (IC₅₀ = 16.77 μmol/L), have been identified as direct ACE inhibitors that interact with the enzyme’s active site.

In amaranth, digestion exerts a more dynamic effect. Gastric treatment improves ACE inhibition (IC₅₀ ≈ 0.039 mg/mL) compared to the native protein (IC₅₀ ≈ 0.079 mg/mL), but intestinal digestion slightly reduces potency (IC₅₀ ≈ 0.081–0.088 mg/mL). This suggests that the initially released peptides are partially degraded (15). Nevertheless, low-molecular-weight (<5,000 Da) fractions retain relevant activity. Enzymatic pre-hydrolysis using alcalase–neutrase can generate peptides that remain stable after SGID (IC₅₀ ≈ 0.31 mg/mL), indicating enhanced gastrointestinal resistance (13).

Processing conditions and physiological digestion models may also affect antihypertensive outcomes. Fermentation alone may reduce post-digestive ACE inhibition in whole quinoa flours due to over-hydrolysis, whereas combining fermentation with thermal treatment increases inhibition to 60–80%, possibly via Maillard-derived compounds (78). Furthermore, ACE inhibitory activity decreases by 10–30% in an older adult digestion model compared to a standard adult model, which highlights the influence of physiological variability on peptide stability and activity.

Overall, antihypertensive activity after digestion reflects a balance between peptide release and degradation; however, this balance is affected by differences in digestion protocols, molecular size, substrate form and assay conditions.

e Antimicrobial, Anti-adipogenesis and Hypoallergenic activities After Digest.

Although antioxidant and enzyme-inhibitory activities have been extensively investigated, comparatively few studies have addressed the additional bioactivities that emerge following simulated gastrointestinal digestion. These effects, which encompass antimicrobial, anti-adipogenic and hypoallergenic properties, are considered together due to the limited and heterogeneous evidence base for each effect.

Bioactive peptides derived from cañihua proteins have been reported to exhibit antimicrobial activity following enzymatic digestion. Specific hydrolysed fractions inhibited microbial growth by ≥45% under *in vitro* conditions. After 4 h of hydrolysis, the glutelin fraction from the Cupi-Sayhua variety inhibited *E. coli*, *C. albicans*, and *S. aureus* by 88.7, 60.3, and 49.3% respectively, and further purification increased inhibition to 95, 70, and 52% (17). The predominance of anionic peptides suggests that charge-dependent interactions with microbial membranes may alter permeability and cell integrity; however, antimicrobial efficacy varies according to varietal origin and fractionation strategy.

In addition to their antimicrobial effects, quinoa hydrolysates derived from digestion have demonstrated the ability to inhibit adipocyte differentiation in 3 T3-L1 cells, suggesting the potential to modulate lipid accumulation and adipogenic regulatory pathways *in vitro* (60).

With regard to allergenicity, simulated digestion produced peptide fractions that showed no detectable IgE reactivity in western blot assays using sera from individuals with a history of cereal allergy (125). This suggests that immunoreactivity is reduced under experimental conditions (Table 4).

**Table 4:** Computational identification, target prediction and experimental validation of bioactive peptides derived from Andean grains.

Source	Peptide	Bioactivity	Target molecule (PDB ID)	Computational tools	Docking score/interaction energy	Experimental validation	Reference
Lupin	AVPFWM; YSGWLGL; AHAGFGMLY	ACE inhibitory	ACE (1O8A)	AutoDock Vina; Discovery Studio	AVPFWM: −9.2; YSGWLGL: −10.3; AHAGFGMLY: −9.2	*In vitro* (ACE IC₅₀): AVPFWM, 6.38 μM; YSGWLGL, 0.14 μM; AHAGFGMLY, 0.69 μM	(109)
Quinoa	IPPG	ACE inhibitory	ACE (1O8A)	AutoDock Tools; PyMOL; Discovery Studio Visualizer	IPPG: −13.63; Lisinopril: −11.81	Candidate peptides identified by in silico screening	(11)
Quinoa	MAF; NMF; HPF; MCG	ACE/DPP-IV inhibitory	ACE; DPP-IV	BIOPEP; PeptideRanker	Candidate peptides identified by in silico screening	*In vitro* (ACE IC₅₀): MCG, 6.48 μg/mL; HPF, 40.08 μg/mL; MAF, 55.93 μg/mL; NMF, 62.34 μg/mL. In vitro DPP-IV inhibition (IC₅₀): HPF, 13.69 μg/mL; MCG, 45.95 μg/mL; NMF, 52.26 μg/mL; MAF, 124.35 μg/mL	(93)
Quinoa	NIFRPFAPEL; AALEAPRILNL	ACE inhibitory	ACE (6H5W)	ChemDraw; Chem3D; Schrödinger Suite (Glide); PyMOL	NIFRPFAPEL: −14.72; AALEAPRILNL: −13.50	*In vitro* (ACE IC₅₀): NIFRPFAPEL, 49.02 μM; AALEAPRILNL, 79.72 μM	(76)
Quinoa	PSF; IPG; SPR; CSPG; PPN	ACE inhibitory	ACE (1O8A)	Chimera; AutoDockTools; PyMOL; Discovery Studio	PSF: −11.15; IPG: −7.69; SPR: −7.81; CSPG: −10.47; PPN: −11.03; Lisinopril: −11.81	Not experimentally validated	(134)
Quinoa	PSF; IPG; SPR; CSPG; PPN; SPF	DPP-IV inhibitory	DPP-IV (1×70)	Chimera; AutoDockTools; PyMOL; Discovery Studio	PSF: −8.99; IPG: −6.43; SPR: −6.54; CSPG: −7.14; PPN: −6.79; SPF: −6.99; Sitagliptin: −8.62	Not experimentally validated	(134)
Amaranth and Quinoa	IW; PW	DPP-IV inhibitory	DPP-IV (1WCY)	Discovery Studio; MGLtools; AutoDock Vina	IW: −8.2; PW: −7.8	Not experimentally validated	(123)
Lupin	AVPFWM; YSGWLGL; AHAGFGMLY	DPP-IV inhibitory	DPP-IV (1×70)	AutoDock Vina; Discovery Studio	AVPFWM: −6.4; YSGWLGL: −8.1; AHAGFGMLY: −6.5	*In vitro* (DPP-IV IC₅₀): AVPFWM, 133.0 μM; YSGWLGL, 16.4 μM; AHAGFGMLY, 1140.0 μM	(109)
Quinoa	IPPG; SPH	DPP-IV inhibitory	DPP-IV (1×70)	AutoDock Tools; PyMOL; Discovery Studio Visualizer	IPPG: −7.80; SPH: −7.86; Sitagliptin: −8.62	*In vitro* (DPP-IV IC₅₀): SPH, 0.54 mg/mL. IPPG: Not experimentally validated (peptide not synthesized).	(11)
Quinoa	IPI; IPV	DPP-IV inhibitory	DPP-IV (1WCY)	Discovery Studio; CDOCKER protocol	IPI: −71.32; IPV: −58.86 (CDOCKER interaction energy)	*In vitro* (DPP-IV IC₅₀): IPI, 5.25 μM; IPV, 26.15 μM. Caco-2 assay: IPI, 10.75 μM; IPV, 29.11 μM	(61)
Quinoa	QHPHGLGALCAAPPST (representative of 35 peptides)	ACE/DPP-IV/α-glucosidase inhibitory	ACE (1O8A); DPP-IV (4A5S); α-glucosidase (5NN3)	Pepsite2	Predicted to bind the active sites of all three target enzymes	Not experimentally validated	(107)
Amaranth and Quinoa	IW; PW	α-Amylase inhibitory	Pancreatic α-amylase (3BAJ)	Discovery Studio; MGLtools; AutoDock Vina	PW: −8.2; IW: −7.7	Not experimentally validated	(123)
Amaranth and Quinoa	IW; PW	α-Glucosidase inhibitory	Maltase-glucoamylase (2QMJ)	Discovery Studio; MGLtools; AutoDock Vina	IW: −7.5; PW: −7.4	Not experimentally validated	(123)
Quinoa	HVASGAGPW; AHCGGLPY; MFVPVPH	Cholesterol inhibitory	Human CEase (1F6W); Bovine CEase (1AQL)	Pepsite 2; Schrödinger Suite	Human CEase: HVASGAGPW, −9.07; AHCGGLPY, −8.95. Bovine CEase: AHCGGLPY, −10.05; MFVPVPH, −9.83	Not experimentally validated	(58)
Quinoa	HVASGAGPW; AHCGGLPY; FSAGGLP	Pancreatic lipase inhibitory	Human PL (1LPB); Porcine PL (1ETH)	Pepsite 2; Schrödinger Suite	Human PL: HVASGAGPW, −11.74; AHCGGLPY, −11.73. Porcine PL: AHCGGLPY, −10.95; HVASGAGPW, −10.19	Not experimentally validated	(58)
Quinoa	FHPFPR; NWFPLPR; HYNPYFPGGA	Anti-cancer	HDAC1 (4BKX)	AutoDock Vina; AutoDock Tools; MGL Tools	FHPFPR: −6.56; NWFPLPR: −6.48; HYNPYFPGGA: −6.41	Not experimentally validated	(35)
Amaranth and Quinoa	IW; PW	α-Glucosidase inhibitory	Sucrase-isomaltose (3LPP)	Discovery Studio; MGLtools; AutoDock Vina	IW: −7.5; PW: −7.4	Not experimentally validated	(123)
Quinoa	AVRFTK; LKTVVVVPG; LLVLL; LKTLLAKP; VVKTLVRP	Selenium-chelating and Antioxidant	Sodium selenite (PubChem).	PyMOL; Discovery Studio (CDOCKER)	AVRFTK: −7.91; LKTVVVVPG: −6.99; LLVLL: −6.06; LKTLLAKP: −5.51	Indirect validation: UV, FTIR, SEM and amino acid composition analysis	(133)
Lupin	EEEEEEPR; EEEEEDEPR; NQLDPSPR; LDPNPR	Iron-chelating	Fe2+ / Fe3 + ions	Discovery Studio Visualizer; Avogadro; AutoDock 4; AutoDockTools; PyMOL	EEEEEEPR: −0.48; EEEEEDEPR: −0.48; NQLDPSPR: −0.42; LDPNPR: −0.41	*In vitro* (Ferrozine assay): EEEEEEPR and EEEEEDEPR showed the highest iron-chelating activity ~0.4 μg Fe2+/mg)	(130)
Quinoa germinated	NLFRP; WDLRLP; ERDPF; FRLEP	ACE inhibitory	ACE (1O8A)	PeptideRanker; ToxinPred; ADMETlab 3.0; RDKit; Boruta; Pre-GRU; PaddleHelix; PyMOL; Discovery Studio	NLFRP: −9.60; WDLRLP: −10.20; ERDPF: −9.80; FRLEP: −9.80	*In vitro* (ACE IC₅₀): NLFRP, 3.33 μM; inhibition kinetics	(67)
Quinoa beverage	SCAWLLAWSAPK; SLSALLQLIR	Anti-inflammatory	TLR4 / MD2 / LPS complex (3FXI)	ToxinPred; PeptideRanker; PreAIP; PEP-FOLD4; AutoDock Vina; Discovery Studio; PyMOL	SCAWLLAWSAPK: −8.5; SLSALLQLIR: −8.5	Cell-based assay: inhibition of NO, IL-6, and TNF-*α* production	(127)
Quinoa (11S globulin)	NIYQIS; QDQHQKIR; YDDER	ACE, DPP-IV, α-amylase, α-glucosidase inhibitory; Anti-inflammatory	α-amylase (4 W93); α-glucosidase (3 W37); DPP-IV (2P8S); INSR (1IRK); ACE-I (1O8A); Lipoxygenase (1N8Q)	BIOPEP; PeptideRanker; ToxinPred; Peptide 2.0; ADMETlab 3.0; PepBDB; PyMOL; UCSF Chimera; PDBsum; VMD; NAMD (Molecular Dynamics Simulations); Carma	NIYQIS: −158.2 to −205.0; QDQHQKIR: −161.5 to −219.3	In vitro enzymatic assays: NIYQIS showed the highest ACE, DPP-IV and ORAC activity; QDQHQKIR showed the highest α-amylase inhibition and Cu^2+^-chelating activity	(108)
Quinoa	APELLSEAFDVPEDLIRK	α-Amylase/α-glucosidase inhibitory; Antioxidant	α-amylase (1PIF); α-glucosidase (5ZCB); Pubchem: structures of DPPH (CID: 2735032) and ABTS (CID:5360881)	AutoDock Vina; PyMOL	α-Amylase: −176.4; α-glucosidase: −89.1; DPPH: −3.56	Not experimentally validated	(55)
Quinoa	IDL; VSF; IEL; EVF	ACE inhibitory	ACE (1O8A)	BIOPEP (PeptideCutter); RDKit; OpenBabel; K-means clustering; AutoDock Vina; PyMOL; PLIP	IDL: −10.58; VSF: −10.44; EVF: −10.57; IEL: −10.23	*In vitro* (ACE IC₅₀): VSF, 15.4 μM; IDL, 65.2 μM. Cell assay (EA.hy926) and *in vivo* (SHR rats)	(128)
Quinoa	IQAEGGLT (parent); RQAEGRLT; KQAEGKLT; HQAEGHLT	α-Amylase/α-glucosidase inhibitory	α-Amylase (1PIF)	PEP-FOLD4; PepSite2; HADDOCK 2.4; CABS-dock; PRODIGY; HawkDock (MM/GBSA); YASARA (MD/MM-PBSA); BIOVIA	RQAEGRLT showed the highest predicted binding affinity	*In vitro* (α-amylase IC₅₀): RQAEGRLT, 9.75 μM	(129)

ACE, angiotensin-converting enzyme; DPP-IV, dipeptidyl peptidase IV; CEase, cholesterol esterase; PL, pancreatic lipase; HDAC1, histone deacetylase 1; INSR, insulin receptor; TLR4, Toll-like receptor 4; MD2, myeloid differentiation factor 2; LPS, lipopolysaccharide; NO, nitric oxide; IL-6, interleukin-6; TNF-α, tumor necrosis factor alpha; ORAC, oxygen radical absorbance capacity; FTIR, Fourier-transform infrared spectroscopy; SEM, scanning electron microscopy; UV, ultraviolet spectroscopy; IC₅₀, half-maximal inhibitory concentration; PDB, Protein Data Bank; MD, molecular dynamics; MM/GBSA, Molecular Mechanics/Generalized Born Surface Area; MM/PBSA, Molecular Mechanics/Poisson–Boltzmann Surface Area; SHR, spontaneously hypertensive rats.

Taken together, these underexplored bioactivities broaden the functional scope of digestion-derived peptides from Andean grains. However, firm conclusions regarding physiological relevance are limited by the small number of independent studies, the predominance of in vitro models, and the lack of standardized methodologies.

## 6 In silico approaches for the identification and characterization of bioactive peptides from Andean grains

Computational approaches have become invaluable in accelerating the discovery and characterization of bioactive peptides from food proteins. They facilitate rapid screening of peptide sequences, prediction of biological potential, evaluation of physicochemical and pharmacokinetic properties, and investigation of peptide–target interactions prior to experimental validation, thereby reducing the time and cost associated with peptide discovery. More recently, deep learning and machine learning approaches have made an even greater contribution to peptide prioritization and bioactivity prediction (32, 51, 58), complementing conventional computational workflows (126–128).

Several complementary bioinformatics tools have been used to identify bioactive peptides in Andean grains. BOPEP-UWM and PeptideRanker are widely used to simulate enzymatic hydrolysis and prioritize potentially bioactive peptides, whereas PepSite2, ToxinPred and ADMETlab, were employed to predict peptide-protein interactions, toxicity and pharmacokinetic properties before the molecular docking analyses (32, 51, 58, 126, 127, 129). Despite these advances, computational predictions are still dependent on the assumptions underlying individual algorithms. Therefore, they should be regarded as supplementary evidence that requires experimental confirmation in order to verify peptide stability, bioavailability and biological activity under physiological conditions (32, 58, 126, 130).

Among the available computational approaches, molecular docking remains the predominant approach for investigating peptide–target interactions because it provides structural insights into binding affinity and potential mechanisms of action before experimental validation (11, 131, 132). For example, several studies used AutoDock Vina or similar docking platforms (11, 58, 96, 123). Also, recent investigations have adopted additional approaches, such as CDOCKER, HADDOCK and molecular dynamics simulations, to improve the reliability of predictions (108, 129, 133). Docking results are generally ranked according to their predicted binding affinity, with more favorable scores suggesting stronger peptide–target interactions. However, these scores are not directly comparable across studies because different docking algorithms and scoring functions are used, and predicting the effects of peptide flexibility remains challenging (132). The principal computational approaches used for the identification and characterization of bioactive peptides derived from Andean grains, together with their corresponding experimental validation, are summarized in Table 5.

**Table 5:** *In vivo* studies of bioactive peptides from Andean grains.

Treatment	Bioactive Activity	Andean grain	Source / Fraction	Model	Assessment method	Main result	Source
Hydrolysis (Alcalase) + Ultrafiltration	Immunomodulatory	Quinoa	Peptide fraction (<3,000 Da)	BALB/c mice	*Ex vivo* phagocytosis assay	Oral dose (1,000 μg/mL) increased phagocytic activity by 39%, IL-10 stimulation	(52)
Hydrolysis (pepsin-trypsin)	Antifatigue	Quinoa	Protein hydrolysate	C57BL/6 J mice	Weight-loaded swimming test	QPH prolonged swimming time, increased muscle glycogen (~1.54 mg/g), and regulated mitochondrial AMPK/PGC-1α pathway.	(38)
Hydrolysis (pepsin-pancreatin)	Anti-inflammatory (Colitis)	Quinoa	Peptide fraction (<3,000 Da)	C57BL/6 J mice	DAI score; Histology; Western blot	Alleviated colitis symptoms, inhibited TLR4/NF-κB pathway, and reduced serum inflammatory cytokines (TNF-α, IL-6).	(34)
Hydrolysis (pepsin-trypsin)	Anti-obesity	Quinoa	Peptide fraction (<3,000 Da)	C57BL/6 J mice	Body weight; Lipid profile	Reduced body weight gain by 16.67% and regulated hepatic lipid metabolism via PPAR-α/γ signaling.	(135)
Hydrolysis (Alcalase) + EDUF fractionation	Antihypertensive	Quinoa	Neutral Fraction	SHR rats	Systolic blood pressure	Oral administration (100 mg/kg) reduced SBP by -29.10 mmHg after 6 h (Control Captopril: -17.49 mmHg).	(31)
Extrusion	Anti-anemic	Quinoa (Negra Collana)	Extruded Flour	Anemic rats	Hematocrit levels	Oral dose (360 mg/Kg) increased hematocrit to 53.8%.	(136)
Extrusion	Anti-anemic	Cañihua (Ramis)	Extruded Flour	Anemic rats	Hematocrit levels	Oral dose (360 mg/Kg) increased hematocrit to 51.7%.	(136)
SGID (pepsin-pancreatin)	Antihypertensive	Quinoa	Protein hydrolysate	SHR rats	Blood pressure (Tail-cuff)	Oral dose (400 mg/kg) reduced systolic blood pressure by -33.1 mmHg (6 h), comparable to Captopril (-35 mmHg).	(93)
SGID (pepsin-trypsin) + Ultrafiltration (<3,000 Da)	Anti-inflammatory	Quinoa	Peptide fraction	DSS mice	DAI score; Cytokines	Peptide alleviated colitis, reduced TNF-α, IL-6 and suppressed the NF-kB pathway.	(34)
SGID (pepsin-trypsin) + Ultrafiltration (<3,000 Da)	Anti-obesity	Quinoa	Peptide fraction	HFD mice	Body Weight; Adipose Tissue	Peptide (1,000 mg/kg) reduced body weight gain (16.67%) and epididymal fat.	(135)
Hydrolysis (Pepsin)	Antioxidant and Anti-aging	Quinoa	Protein hydrolysate	D-galactose-induced aging mice	Organ indices, biochemical assays (SOD, CAT, GSH-Px, T-AOC, MDA), qRT-PCR, and Western blot	Reversed thymus/spleen atrophy, reduced MDA, and upregulated SOD1, SOD2, CAT, and GSH1 gene expression.	(54)
SGID (Pepsin + Trypsin) + Nano-LC–MS/MS sequencing + Database prediction (PeptideRanker)	Anti-inflammatory and mucus barrier protection	Quinoa	Chemically synthesized pure peptide (TPGAFF)	C57BL/6 J mice with DSS-induced colitis	Disease Activity Index, H&E and Alcian blue staining, ELISA, RT-qPCR, and 16S microbiota sequencing	Oral dose (50 and 150 mg/kg/day for 28 days) attenuated colitis phenotype, restored mucus barrier, inhibited NF-κB-TRPV1 signaling, and positively regulated gut microbiota	(140)

AMPK, AMP-activated protein kinase; CAT, catalase; DAI, Disease Activity Index; DSS, dextran sulfate sodium; EDUF, electrodialysis with ultrafiltration; ELISA, enzyme-linked immunosorbent assay; GSH-Px, glutathione peroxidase; H&E, hematoxylin and eosin; HFD, high-fat diet; IBD, inflammatory bowel disease; IL-6, interleukin-6; IL-10, interleukin-10; MDA, malondialdehyde; NF-κB, nuclear factor kappa B; PGC-1α, peroxisome proliferator-activated receptor gamma coactivator 1-alpha; PPAR-α, peroxisome proliferator-activated receptor alpha; PPAR-γ, peroxisome proliferator-activated receptor gamma; QPH, quinoa protein hydrolysate; qRT-PCR, quantitative reverse transcription polymerase chain reaction; SBP, systolic blood pressure; SGID, simulated gastrointestinal digestion; SHR, spontaneously hypertensive rats; SOD, superoxide dismutase; T-AOC, total antioxidant capacity; TLR4, Toll-like receptor 4; TNF-α, tumor necrosis factor alpha; TRPV1, transient receptor potential vanilloid 1.

ACE and DPP-IV are predominant molecular targets investigated in Andean grain-derived peptides. ACE-inhibitory peptides identified in quinoa, amaranth and lupin typically contain 3–16 amino acid residues, with docking energy values between −7.0 to −14.72 kcal/mol. Some peptides have demonstrated stronger predicted affinities than the reference inhibitor, lisinopril (−11.81 kcal/mol) (11, 76, 93, 96). Likewise, DPP-IV inhibitory peptides exhibited docking energies between −6.4 to −9.0 kcal/mol, values comparable to those reported for sitagliptin (−8.62 kcal/mol) (134). Furthermore, several peptides selected through computational modeling were subsequently validated through experimentation, including chemically synthesized ACE inhibitors, as well as recently identified peptides such as NLFRP (IC₅₀ = 3.33 μM), VSF (IC₅₀ = 15.4 μM) and IDL (IC₅₀ = 65.2 μM) (126, 128). Similarly, the DPP-IV inhibitory peptides IPI and IPV demonstrated strong interaction energies and retained inhibitory activity following validation in enzymatic assays and Caco-2 cell models (61). These findings collectively indicate that computational screening has evolved from a simple prioritization strategy based on docking scores into an effective method of identifying peptides with confirmed biological activity.

A clear trend emerging from recent studies is the diversification of computational targets beyond ACE and DPP-IV toward the identification of multifunctional peptides. Besides enzymes involved in blood pressure and glucose regulation, peptides have been predicted to interact with *α*-amylase, α-glucosidase, maltase-glucoamylase, sucrase-isomaltase, cholesterol esterase, pancreatic lipase, HDAC1, TLR4/MD2 and mineral-chelating systems for iron and selenium (35, 55, 58, 108, 123, 130, 133). This diversification might reflect a conceptual shift from searching for peptides with isolated biological activities toward multifunctional sequences capable of simultaneously modulating multiple metabolic and inflammatory pathways also, these developments demonstrate how computational methods have evolved from virtual screening to integrated platforms for peptide design and optimization.

Another important trend emerging from recent studies is the progressive integration of computational prediction and experimental validation. While earlier investigations primarily ended at molecular docking, contemporary workflows increasingly incorporate peptide synthesis, enzymatic assays, cell-based models and, more recently, animal experiments. However, analysis of the available literature suggests that only a small proportion of computationally predicted peptides have progressed beyond *in vitro* enzymatic assays. Validation in cellular systems is limited, and *in vivo* confirmation is restricted to a few candidates (126, 128). This translational gap is one of the main challenges in computational peptide discovery, highlighting the need for standardized pipelines that integrate simulated gastrointestinal digestion, molecular docking, molecular dynamics simulations and multi-level biological validation. Such integrated strategies will improve the reliability of peptide selection and accelerate the translation of computational predictions into clinically relevant functional foods and nutraceuticals.

## 7 *In vivo* studies of bioactive peptides in Andean grains

Preclinical studies, predominantly involving quinoa-derived peptides, suggest pleiotropic physiological effects in animal models of metabolic disorders, inflammation, cardiovascular disease, oxidative stress, and physical fatigue. However, the available evidence remains limited, with most studies focusing on quinoa and only a few evaluating other Andean grains. A summary of the current *in vivo* evidence is presented in Table 5.

Regarding metabolic disorders, Busso et al. (32) reported that oral administration of quinoa protein hydrolysates in mouse models of gestational diabetes mellitus prevented glucose intolerance and reduced fetal weight and hepatic triglyceride accumulation. These effects were associated with improved hepatic insulin signaling through activation of the downstream p-AKT and p-MAPK pathways (32). Similarly, quinoa peptide fractions significantly reduced body weight gain, hepatic lipid accumulation, and systemic inflammation in high-fat diet-induced obese mice, as evidenced by lower serum TNF-*α* and IL-6 concentrations (34). Furthermore, quinoa peptides restored the *Firmicutes/Bacteroidetes* ratio and increased the abundance of *Muribaculaceae*, suggesting that modulation of the gut microbiota may contribute to the metabolic improvements observed in obese mice (135).

Protective effects have also been observed in gastrointestinal health and immune regulation. In experimental colitis models, quinoa peptides (< 3,000 Da) reduced intestinal mucosal damage, attenuated inflammatory responses, promoted short-chain fatty acid production, and restored gut microbial diversity (34). In addition, Wang et al. (136)showed that the oral administration of specific, chemically synthesized quinoa peptide TPGAFF (50 and 150 mg/kg/day for 28 days) attenuated disease severity in DSS-induced colitis mice. The peptide also protected intestinal barrier integrity and promoted a healthier gut microbial composition. Furthermore, quinoa peptide fractions may exert systemic immunomodulatory effects, even in healthy subjects, improving splenic macrophage phagocytosis while maintaining immune homeostasis through coordinated regulation of IL-10, TNF-*α*, and IFN-*γ* (38).

Regarding cardiovascular health, oral administration of SGID-derived peptides produced acute and sustained reductions in blood pressure in spontaneously hypertensive rats, with antihypertensive effects comparable to those of captopril, an effect that has been attributed to ACE inhibition (30, 31). More recently, Zhang et al. (128) reported that the oral administration of the quinoa-derived tripeptides IDL and VSF (30 mg/kg for 4 weeks) significantly reduced systolic and diastolic blood pressure in spontaneously hypertensive rats. Beyond their antihypertensive activity, these peptides were also associated with reduced vascular fibrosis and protection of cardiac and renal tissues, suggesting broader cardioprotective effects.

Quinoa proteins and their hydrolysates have also been associated with improvements in physical performance and resistance to exercise-induced fatigue. Animals receiving these treatments exhibited greater endurance together with lower blood lactate, blood urea nitrogen and creatine kinase concentrations, indicating improved recovery after physical exertion. While these effects have been linked to changes in gene expression, the specific peptides responsible and the underlying molecular mechanisms still require characterization and validation (38). Similarly, Gao et al. (54) reported that oral supplementation with pepsin-derived quinoa peptides effectively attenuated systemic oxidative stress in a D-galactose-induced aging mouse model. The treatment enhanced endogenous antioxidant defenses, reduced oxidative damage, and protected multiple organs from age-related injury.

Evidence from other Andean grains is limited. In rat models of iron-deficiency anemia, oral administration of extruded quinoa (Negra Collana) and cañihua (Ramis) flours (360 mg/kg) significantly increased hematocrit levels and hemoglobin levels, suggesting anti-anemic activity (136). Nevertheless, studies evaluating purified bioactive peptides from cañihua, amaranth, and lupin are still largely unavailable.

Overall, the available *in vivo* evidence suggests that bioactive peptides derived from Andean grains, especially quinoa, may contribute to the regulation of metabolism, inflammation, cardiovascular function, oxidative stress and immune homeostasis. Recent studies have progressed from evaluating crude hydrolysates to investigating chemically synthesized and structurally characterized peptides, providing stronger evidence supporting peptide-specific biological activities. Nevertheless, evidence remains largely restricted to quinoa, highlighting the need for further *in vivo* studies in other Andean grains. Current knowledge is still largely based on relatively short-term rodent studies. Therefore, additional long-term animal studies and well-designed human clinical trials are required to confirm the bioavailability, safety and optimal dosage of peptides, as well as their therapeutic efficacy.

## 8 Application of bioactive peptides from Andean grains in food formulation

**Figure 3:**
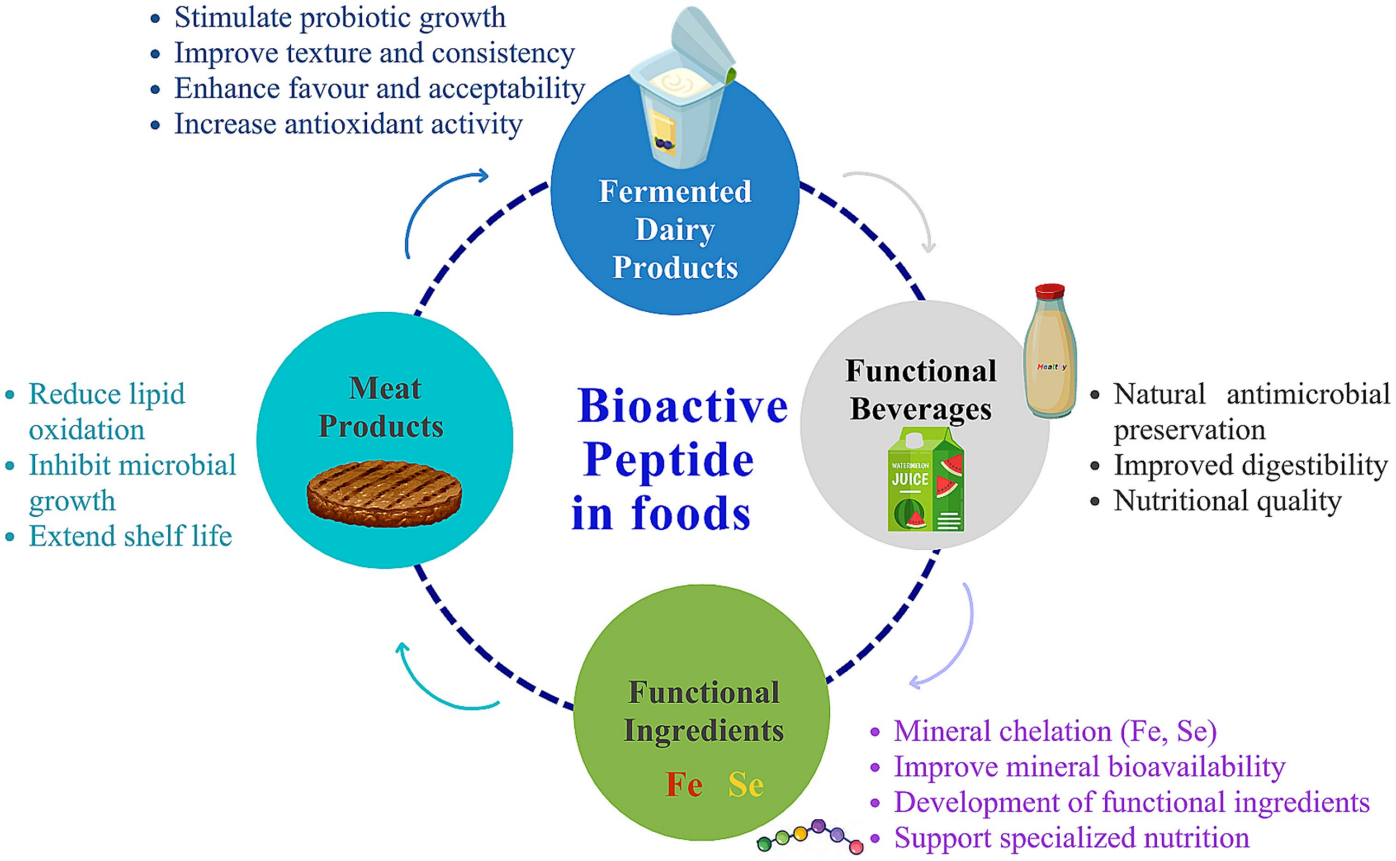
Potential applications of bioactive peptides derived from Andean grains in food systems.

Despite the growing evidence on the health benefits of bioactive peptides derived from Andean grains, their application in food systems remains at an early stage and is largely restricted to laboratory-scale prototypes (Figure 3). Current strategies mainly involve the incorporation of protein hydrolysates or purified peptides into fermented or processed foods to improve nutritional quality, technological performance, microbial stability, sensory attributes, and functional properties.

### 8.1 Dairy products

In dairy matrices, Chen et al. (137) demonstrated that the addition of quinoa protein hydrolysates (1–2% w/v) to yogurt fermented with *Lactobacillus plantarum* significantly stimulated bacterial growth and the production of organic acids, including lactic and citric acids. Hydrolysate supplementation also improved water-holding capacity, viscosity, and antioxidant activity. Moreover, supplementation at 2% enhanced the formation of 52 volatile compounds, resulting in a more desirable flavor profile and higher consumer preference (137). Collectively, these findings suggest that quinoa-derived peptides may function as multifunctional food ingredients by simultaneously improving the technological, sensory, and functional properties of fermented dairy products.

### 8.2 Beverages

Beyond dairy products, quinoa-derived peptides have also shown promising applications in beverage formulation. Zhu et al. (36) explored the application of purified quinoa peptide fractions (< 1,000 Da) in fresh apple juice. These peptides effectively suppressed spoilage microorganisms such as *Pantoea* and *Leuconostoc*, while improving juice clarity, increasing soluble solids and titratable acidity, and promoting the growth of *Gluconobacter*, a beneficial bacterium with probiotic potential. Notably, the peptide AGAAPE exhibited excellent thermal and pH stability, resistance to ionic interference (Na^+^, Ca^2+^) and enzymatic hydrolysis, highlighting its potential as a natural antimicrobial preservative (36). However, the purification procedures required to obtain individual peptides may increase production costs and limit their industrial scalability.

Quinoa beverages have also attracted attention because of their nutritional quality. Zhao et al. (89) reported that quinoa-based beverages exhibited significantly higher *in vitro* protein digestibility compared to soy, chickpea, pea, and oat-based alternatives, highlighting the high protein quality and digestibility of quinoa-based beverages (89). Building on theses nutritional advantages, Cirat et al. (138) formulated a fermented quinoa beverage with a high Digestible Indispensable Amino Acid Score (DIAAS) (138). Therefore, this could support its potential application in specialized nutritional products and functional beverages.

### 8.3 Meat products

In meat products, Yekta et al. (139) showed that liposome- encapsulated quinoa peptides (5 mg/mL) improved the oxidative and microbial stability of hamburgers during 12 days of refrigerated storage. The treated samples exhibited reduced lipid oxidation, protein degradation, and microbial growth, including *Staphylococcus* and yeasts (139). These findings suggest that quinoa-derived peptides may serve as natural preservatives capable of extending the shelf life of meat products. However, the absence of sensory evaluation and validation under industrial processing conditions limits conclusions regarding consumer acceptance and the practical applicability of these formulations.

### 8.4 Functional ingredients

Quinoa is not the only Andean pseudocereal showing promise as a source of functional ingredients. Montoya-Rodríguez et al. (84) highlighted that extruded amaranth flours are naturally enriched in peptides with ACE and DPP-IV inhibitory activities, supporting their direct incorporation into food formulations or dietary supplements aimed at promoting metabolic health (84).

Beyond their direct incorporation into foods, advanced delivery systems are being explored to improve peptide stability and functionality. Intiquilla et al. (91) successfully nano encapsulated antioxidant peptide fractions (<3,000 Da) obtained from lupin in chitosan nanoparticles, demonstrating a promising strategy to protect peptides during gastrointestinal transit and potentially enhance their bioavailability (91).

Recent studies have expanded the use of Andean grain peptides as mineral delivery systems even further. For example, Chai et al. (133) developed a quinoa peptide–selenium chelates and found that fractions of the peptide with a molecular weight of less than 3,000 Da had a high capacity to chelate selenium, strong antioxidant activity and excellent stability across a wide range of pH and temperature conditions. These findings their potential as functional selenium supplements. Similarly, Ranilla et al. (130)demonstrated that the simulated gastrointestinal digestion of *Lupinus mutabilis* proteins released iron-chelating peptides that had a higher iron-binding capacity than casein-derived peptides. Chemically synthesized glutamic acid-rich peptides confirmed this activity, suggesting that they could be used as functional ingredients to improve iron nutrition.

While these studies demonstrate the versatility of Andean grain peptides as multifunctional food ingredients and nutrient delivery systems, most applications are still in the proof-of-concept stage. Further research is needed to address issues such as peptide stability during food processing, gastrointestinal bioavailability, production costs, regulatory requirements and industrial-scale manufacturing before commercial implementation can be achieved.

## 9 Future perspectives on the application of bioactive peptides

Despite the rapid growth of research into Andean grain-derived bioactive peptides, the number of products available commercially, such as functional foods, nutraceuticals, and therapeutic products, remains limited. While many peptides have shown promising *in vitro* bioactivities, few have advanced to preclinical evaluation, industrial development or clinical validation. Overcoming this challenge will require multidisciplinary efforts, integrating food science, nutrition, biotechnology, bioinformatics and clinical research.

One of the major challenges is understanding the fate of bioactive peptides following oral administration. In order to exert their physiological effects, bioactive peptides must remain sufficiently stable enough to resist extensive proteolytic degradation during gastrointestinal digestion and reach their target tissues following intestinal absorption. Therefore, future studies should evaluate gastrointestinal stability, bio accessibility, intestinal absorption, metabolism, pharmacokinetics and dose–response relationships, using standardized digestion models, long-term animal studies and well-designed clinical trials.

From a technological perspective, improving the scalability and reproducibility of peptide production remains essential. Standardized hydrolysis and purification protocols, together with emerging processing technologies such as high hydrostatic pressure, ultrasound, pulsed electric fields and controlled fermentation, could enhance both the yield and functionality of peptides. Similarly, encapsulation technologies offer promising opportunities to improve peptide stability, bioavailability and sensory quality, while also facilitating industrial application.

In addition, the successful commercialization of bioactive peptides will require compliance with the regulatory frameworks that govern health claims, safety assessments, manufacturing quality and efficacy. Standardized validation protocols and harmonized regulatory guidelines will be essential to facilitate the development of functional foods and nutraceutical products containing these peptides.

Future research should also expand beyond quinoa to include other Andean grains that are not as well-known, such as cañihua, amaranth, and tarwi. Integrating proteomics, peptidomics, metabolomics, artificial intelligence-assisted peptide discovery and standardized biological validation will accelerate the identification of multifunctional peptides and facilitate their development into safe, evidence-based functional foods and nutraceuticals. At the same time, adding value to protein-rich by-products from these crops could contribute to the diversification of agriculture and the development of a sustainable circular bioeconomy.

## 10 Conclusion

Bioactive peptides derived from Andean grains represent a promising and versatile resource with potential applications in health, nutrition, and sustainable development. These peptides have been shown to possess antioxidant, antihypertensive, antidiabetic, anti-inflammatory, antimicrobial, antifatigue, anti-hemolytic and anticancer properties, making them valuable ingredients in the development of safer and more effective health-promoting products.

However, despite the breadth of evidence from *in vitro* and preclinical studies, their use in functional foods and nutraceuticals is still limited. In order to progress, it is crucial to establish standardized methodologies, validate bioactivity through human clinical trials and develop robust regulatory frameworks that can substantiate health claims and guarantee consumer safety.

Underexplored grains such as Lupin, amaranth and cañihua should be given special attention, as they provide unique nutritional and bioactive profiles yet remain insufficiently characterized. Expanding research beyond quinoa would not only diversify applications and support agricultural resilience, but also promote the sustainable use of Andean biodiversity.

Advancing this field ultimately requires a multidisciplinary approach that integrates food science, biotechnology, computational biology and clinical research. Such an integrated approach will accelerate the discovery of novel peptides, enhance their functional application and bridge the gap between basic research and industrial implementation. In this way, peptides derived from Andean grains could play a key role in developing next-generation functional foods and therapeutic solutions that align with global health and sustainability goals.
