# Supplementary material for: Exploring bioactive peptides from Andean grains and their potential applications: a review
Source: Front Nutr. 2026 Aug 5;13:1875238. doi: 10.3389/fnut.2026.1875238 (PMC13485802; doi:10.3389/fnut.2026.1875238)
Supplement: Supplementary file 1 [file Table_1.DOCX]

**Supplementary Table S1.** Extended compilation of studies reporting peptide-generation strategies applied to Andean grains.

| **Andean Grain** | **Protein Source** | **Generation Strategy** | **Enzyme /**  **Process** | **Experimental Conditions** | **Main Results (focus on degree of Hydrolysis)** | **Reference** |
| --- | --- | --- | --- | --- | --- | --- |
| Quinoa | Protein isolate | Enzymatic Hydrolysis | Trypsin;  alcalase | 5% (w/v); E:S ratio 0.5% (w/w); pH 8.0; 40 °C; up to 4 h | DH: trypsin (50.25%) at 4 h > Alcalase (~20.9%); rapid hydrolysis within 30 min | (Abbasi et al., 2022) |
| Quinoa | Protein concentrate | Enzymatic Hydrolysis | Pancreatin | 5% (w/v); E:S ratio 2.5% (w/w); pH 7.4; 40 °C; up to 3 h | DH: maximum 19.17% at 180 min; rapid hydrolysis within first 150 min | (Daliri et al., 2021) |
| Cañihua (Ramis/ Kupis Sayhua) | Protein fractions (albumin, globulin 7S-11S, glutelin) | Enzymatic Hydrolysis | Alcalase  Pepsin → Pancreatin | Alcalase: E:S ratio 1:10–1:50; pH 8.0; 50 °C; 2–24 h; SGID: pepsin (pH 2; 1-4h)  Pancreatin (pH 7.5; 4h) | DH: albumin (67%, highest, SGID); glutelins (40%); Alcalase (7–54%) | (Moscoso-Mujica et al., 2021) |
| Amaranth and Quinoa | Protein isolate | Enzymatic Hydrolysis | Bromelain; chymotrypsin; protease | 4% (w/v); E:S ratio 1:100 (w/w); pH 7.0–8.0; 50 °C; 2-6 h | DH: quinoa: Chymotrypsin (87.5%) > protease > bromelain at 6 h. Amarant: Bromelain (47.2%) > chymotrypsin > protease. | (Mudgil et al., 2019) |
| Quinoa | Protein isolate | Enzymatic Hydrolysis | Bromelain; Chymotrypsin; Pronase E | 4% (w/v); E:S ratio 1:100 (w/w); pH 7.0–8.0; 50 °C; 6 h | DH: Chymotrypsin (88.95%) > pronase E (82.5%) > bromelain (76.1%) at 6 h | (Mudgil et al., 2020) |
| Red Quinoa | Seed protein | Enzymatic Hydrolysis | Thermolysin; pepsin; Trypsin; α-chymotrypsin | E:S ratio 1:50 (w/w) protein; pH 2.0–8.5; 37 °C; 16 h (Thermolysin at 60 °C) | Thermolysin produced peptides <5 kDa more efficiently than other enzymes | (Suleman et al., 2024) |
| Quinoa | Protein concentrate | Enzymatic Hydrolysis | Pepsin;  Pancreatin;  Papain | pH 2.0–7.5; 37–55 °C; up to 120 min  SGID: Pepsin pH 2;  Pancreatin pH 7.5; 37 °C.  Papain (7.0); 55 °C, 120 min | DH: pepsin (~35%) > pancreatin (~20%) > papain (~15%) at 120 min | (Shi et al., 2019) |
| Quinoa | Protein concentrate | Enzymatic Hydrolysis | Neutrase | 2.5% (w/v) protein; pH 7.0; 50 °C; 120 min | N.R. | (Cisneros-Yupanqui et al., 2022) |
| Quinoa | Protein | Enzymatic Hydrolysis | Alcalase  Trypsin  Neutrase, Flavourzyme  Papain | E:S ratio 2:100 (w/w); 45 °C; optimal pH per enzyme; 240 min | DH: Alcalase (~26%) > trypsin > Neutrase > Flavourzyme > papain  Alcalase hydrolysate had a trimodal particle size distribution (10–100 nm) | (Gan et al., 2025) |
| Quinoa | Protein (Alkali extracted) | Enzymatic Hydrolysis  (Optimization) | Compound protease; alcalase; papain  ; Flavourzyme; neutral protease | 4% (w/v); 2000 U/g enzyme; 50 °C; 120 rpm; pH optimized (7-10) | DH: compound protease (22.0%) > alcalase (19.9%) > papain (10.1%) > flavourzyme (7.7%) > neutral protease (4.5%); Alcalase selected for best antioxidant performance | (Gu et al., 2024) |
| Quinoa | Protein concentrate | Enzymatic Hydrolysis  (Optimization) | Alcalase → Pepsin | Alcalase: pH 8.0, 4 h; Pepsin: pH 2.5; optimization range: 40–55 °C, 60–210 min, E/S 30–90 AU/kg | DH: maximum 24.65% at 55 °C and 210 min; enzyme activity reached 60 AU/kg | (Mahdavi-Yekta et al., 2019) |
| Lupin | Protein isolate | Enzymatic Hydrolysis (Sequential) | Pepsin → pancreatin → alcalase | 4% (w/v);  Pepsin: 50 mg/mL, E:S 1:50 (v/v), pH 1.9, 37 °C, 30 min;  Pancreatin: 50 mg/mL, E:S 1:50 (v/v), pH 7.8, 37 °C, 60 min;  Alcalase: 2.4 U/g, E:S 1:200 (v/v), pH 8.5, 50 °C, 138 min | DH: Pepsin + Pancreatin reached 17.8%; Pepsin + Pancreatin + alcalase reached 46.12% | (Intiquilla et al., 2022) |
| Quinoa and Kiwicha | Protein concentrate | Enzymatic Hydrolysis/sequential hydrolysis | Alcalase  Neutrase  Flavourzyme | 2.5% (w/v) protein  50 °C, 240 min  Enzyme 1: (60–90 min)  Enzyme 2: (up to 240 min)  pH: Alcalase (8.3), Flavouryzyme/ Neutrase (7.0) | DH: Kiwicha: Alcalase-Flavourzyme reached 76.3%. Quinoa: Neutrase single stage reached 36.9%; Alcalase-Flavourzyme reached 61.8%. | (Chirinos et al., 2020) |
| Lupin | Protein concentrate | Enzymatic Hydrolysis | Alcalase → Neutrase | 2.5% (w/v); 50 °C; Alcalase: pH 8.3, 0.385 AU/g, 60 min; Neutrase: pH 7.0, 50 LAPU/g, 120 min | Peptide size: low MW (<3000 Da); short peptides (6–9 residues) identified | (Chirinos et al., 2022) |
| Amaranth | Protein isolate | SGID  +  Enzymatic Hydrolysis | Alcalase  +  Pepsin → Pancreatin | Alcalase (optimized); GI digestion: Pepsin: pH 2, 60 min; Pancreatin: pH 6, 60 min | DH: Alcalase 29.2%; GI digestion 36.9%; Alcalase + GI digestion 42.0% | (Orsini et al., 2015) |
| Amaranth | Unprocessed / Extruded flour | Extrusion  +  SGID | Pepsin → Pancreatin | Extrusion: 125 °C, 130 rpm; Digestion: Pepsin: pH 2, 180 min; Pancreatin: pH 7.5, 180 min | Peptide profile: extrusion promoted release of peptides <1000 Da; maximum DH at 360 min | (Montoya-Rodríguez et al., 2015) |
| Quinoa | Flour | Fermentation | *Lactobacillus plantarum* T0A10 | Dough yield 160; fermentation at 30 °C (16 h) or 37 °C (24 h) | Peptide concentration: increased soluble peptides to 31.5 mg/mL; antioxidant peptides identified | (Rizzello et al., 2017) |
| Quinoa | Flour | Fermentation  +  SGID | *Pleurotus ostreatus* | Fermentation: 14 days, 28 °C; drying: 70 °C; digestion: INFOGEST (standard vs elderly model) | Proteolysis: fermentation + drying increased TCA-soluble peptides by 20% | (Sánchez-García et al., 2024) |
| Quinoa (Red/white/Black) | Seeds | Fermentation  (Solid state) | *Rhizopus oligosporus; Aspergillus oryzae; Neurospora intermedia* | Pre-cooked seeds (<30 °C); fermentation: *R.* *oligosporus* (31 °C, 2–6 d), *A.* *oryzae* (25 °C, 2–6 d), *N.* *intermedia* (30 °C, 3–5 d); inoculum: 10⁴ spores/g | Peptide concentration: increased from 0.23 to 3.48 g/100 g (dry basis); highest hydrolysis with *R. oligosporus* | (Starzyńska-Janiszewska et al., 2019) |
| Quinoa | Quinoa meal | Fermentation + Enzymatic Hydrolysis | Lipase/Amylase → Mixed strains (*B.* *subtilis*, etc.) | Pretreatment: lipase/amylase, 50 °C, 6 h; fermentation: 10⁷ CFU/mL inoculum, 37 °C, 180 rpm, 48 h | Peptide yield: 70.17% (48 h); high proportion of peptides <1000 Da (30.3%) and 1000-3000Da (28.48%) | (Zhu et al., 2025) |
| Quinoa | Protein isolate | Germination  +  SGID | Germination  +  Pepsin → Pancreatin | Germination: 24 h, 25 °C, dark; drying: 50 °C, 12 h; digestion: pepsin (pH 2, 2 h) → pancreatin (pH 7.5, 4 h) | Proteolysis: germination increased hydrolysis (ΔAbs 1.039 vs 0.572 in non-germinated samples) | (Salami et al., 2022) |
| Quinoa (White, Red, Black, Gray) | Protein isolate | Germination  +  Hydrolysis | Pepsin → Trypsin; Neutrase; Flavourzyme; Alcalase; Papain | Germination: 0- 48 h. Hydrolysis:  Pepsin (pH 2,37 °C, 90 min), Trypsin (pH 7,37 °C, 240 min)  E:S 2%.  Neutrase (45 °C, pH 7), Alcalase (50 °C, pH 8), Flavourzyme (50 °C, pH 7) and Papain (55 °C, pH 7) at E:S 4% for 180 min. | DH white quinoa: Pepsin-Trypsin: 27.27%, Neutrase: 23.45%. Flavourzyme: 17.01%, Alcalase: 16.57%, Papain: 5.10%.  Long germination (48 h) reduced DH to 5.38%.  DH Black quinoa: Pepsin-Trypsin 21.03 % | (You et al., 2022) |
| Quinoa | Germinated seed protein | Germination  +  SGID | Endogenous enzymes +  Pepsin → Trypsin | Germination: 25°C, 80% RH, 12-72 h. Digestion:  Pepsin (pH 2.0, 2 h) Trypsin (pH 7.5, 2 h), 37°C, 4% E/S ratio. | Digestibility: high in vitro protein digestibility; peptides <1000 Da ~90% of digests; free amino acids increased up to 672 mg/g | (He et al., 2025) |
| Quinoa (White) | Protein isolate | High-Hydrostatic Pressure (HHP) assisted enzymatic hydrolysis | Alcalase  +  HHP | 5% (w/v); E:S ratio 1:20 (0.14 AU/g); pH 8.0; 50 °C; 15 min; pressure: 200–600 MPa | DH: maximum at 300 MPa (9.87%) vs atmospheric (6.06%); pressures >500 MPa reduced DH | (de Carvalho Oliveira et al., 2024) |
| Quinoa | Protein isolate | High-Intensity Ultrasound (HIU) Pretreatment +  Hydrolysis | Ultrasound  +  Alcalase | Ultrasound: 20 kHz, 200–600 W, 20 min; Hydrolysis: 50 °C, pH 9.0, 5 h; E:S 0.4 g/kg protein | DH: maximum at 400 W; higher power (600 W) reduced DH due to aggregation | (X. Li et al., 2018) |
| Quinoa (11S, 13S) | Protein sequences | In silico | Papain; Ficin; Stem; Bromelain | BIOPEP-UWM database simulation (enzyme action tool) | DH (theoretical): 31.29–52.13%; stem bromelain showed highest DH | (Guo, Richel, et al., 2020) |
| Amaranth and quinoa | Protein sequences | In silico | Pepsin; Trypsin;  Chymotrypsin | BIOPEP-UWM database (enzyme action tool) | Bioactivity: 52 antidiabetic peptides predicted; peptides PW, PF, PPG, PM, SW, IW identified | (Valenzuela Zamudio et al., 2024) |
| Quinoa | Flour | Malting  +  SGID | Malting (Endogenous)  +  Pepsin/Trypsin | Malting: 8–15 °C, 44–52% moisture, 3–7 d; Digestion: INFOGEST | Peptide profile: oligopeptides <20 kDa; DPP-IV inhibitory peptides identified (AFP, HI, HL, RI, RL, IR, LR) | (Kröber et al., 2024) |
| Quinoa (Real) | Fractions (Alb, Glob, Glut, Pro) | SGID | Pepsin → Pancreatin | Gastric: pepsin, pH 2, 3 h; Intestinal: pancreatin, pH 7.5, 24 h; E:S 1:10 | DH: glutelins 38.97% > caseins 33.7% > globulins 26.6%; heating increased globulin DH to 34.7% | (Tavano et al., 2022) |
| Quinoa | Quinoa peptides (from Alcalase) | SGID | Pepsin → Pancreatin | Gastric: pepsin (1%), pH 2.0, 37 °C, 4 h; Intestinal: pancreatin (1.5%), pH 7.0, 37 °C, 6 h | DH: increased ~14% during intestinal phase; peptides <2000 Da (98.4%) | (Gu et al., 2024) |
| Quinoa (Mengli-I) | Protein extract | SGID | Pepsin → Pancreatin | Gastric: pepsin (2000 U/mL), pH 3.0, 120 min; Intestinal: pancreatin (100 U/mL), pH 7.0, 120 min | DH: final 21.92% | (Guo, Hao, et al., 2020) |
| Quinoa | Protein concentrate | SGID | Pepsin → Pancreatin | Gastric: pepsin, pH 3.0, 120 min; Intestinal: pancreatin, pH 7.0, 120 min | Peptide size: <5000 Da fraction mainly responsible for bioactivity; larger peptides also contributed | (Vilcacundo et al., 2017) |
| Kiwicha | Protein concentrate | SGID | Pepsin → Pancreatin | Gastric: pepsin, pH 3.0, 120 min; Intestinal: pancreatin, pH 7.0, 120 min | Bioactivity: proteins degraded into peptides <5000 Da with antioxidant and ACE-inhibitory activity | (Vilcacundo et al., 2019) |
| Quinoa | Whole Quinoa Beverage | SGID | Pepsin → Pancreatin | Gastric: pepsin (2000 U/mL), pH 2.0, 120 min; Intestinal: pancreatin (100 U/mL), pH 7.0, 120 min | Digestibility: 80.49%; peptides <1000 Da (93.84%); free amino acids 211.7 mg/g protein | (Zhao et al., 2024) |

DH, Degree of Hydrolysis; GI, Gastrointestinal; RH, Relative Humidity; MW, Molecular Weight; TCA, Trichloroacetic Acid; dwb, Dry Weight Basis; E:C, Enzyme Concentration; N.R., No Reported.

**Supplementary Table S2**. Extended compilation of in vitro bioactivities reported for peptides obtained through enzymatic hydrolysis of Andean grains.

| **Bioactive Activity** | **Treatment** | **Andean Grain** | **Source / Fraction** | **Model** | **Assessment Method** | **Main result** | **Source** |
| --- | --- | --- | --- | --- | --- | --- | --- |
| Antithrombotic | Hydrolysis  (Alcalase + Trypsin) | Amaranth | Glutelins | - | Thrombin Time assay (TT) | Clotting time: 81.0 s vs control 19.5 s | (Sabbione et al., 2015) |
| Anti-hemolytic | Hydrolysis (Chymotrypsin) | Amaranth | Protein Isolate | Cells (Erythrocyte) | Hemolysis inhibition assay | Hydrolysate at 6 h protected erythrocytes with IC_50_ = 0.00083 mg/mL. | (Mudgil et al., 2019) |
| Antioxidant | Synthesis (from *in silico*) | Amaranth, Quinoa | Synthetic Peptides  (IW, PW) | Cells (Caco-2) | ROS assay | Radical scavenging: SW = 32% and IW = 35 % | (Valenzuela Zamudio et al., 2024) |
| Antioxidant | Sequential Hydrolysis (Pepsin-Pancreatin-Alcalase) | Lupin | Peptide Fraction <3000 Da | - | ORAC assay | Antioxidant activity of 5.02 µmol TE/mg protein. | (Intiquilla et al., 2022) |
| Antioxidant | Optimization (Alcalase) | Lupin | Protein Concentrate | - | ORAC assay | Optimized hydrolysis (E/S 1.72%, 133 min): 3.8 µmol TE/mg protein. | (Intiquilla et al., 2018) |
| Antihypertensive | Fermentation (*L. paracasei*) | Quinoa | Fermentation broth | - | Enzyme inhibition assay (ACE) | 86.50%; peptides NIFRPAPEL and ALAEAPRILNL identified | (S. Li et al., 2022) |
| Antihypertensive | Hydrolysis (Chymotrypsin) | Quinoa | Hydrolysate (6 h) | - | Enzyme inhibition assay (ACE) | IC₅₀ = 0.22 mg/mL | (Mudgil et al., 2020) |
| Antihypertensive | Hydrolysis (Neutrase) | Quinoa | Protein Concentrate | - | Enzyme inhibition assay (ACE) | IC₅₀ = 0.08 mg/mL (lowest value) | (Chirinos et al., 2020) |
| Anti-adipogenesis | Hydrolysis (Pepsin) | Quinoa | Hydrolysate | Cells (3T3-L1 adipocytes) | Lipid accumulation assay | Hydrolysate at 120 min  IC₅₀ = 786.58 μg/mL; inhibited adipocyte differentiation | (Shi et al., 2019) |
| Anticancer | Purification (DEAE Cellulose) | Quinoa | Purified Protein Fraction (pH 2) | Cells (A549, HeLa) | MTT assay | Significant cytotoxicity;  IC₅₀ = 11.21 μg/mL (A549 cells) | (Sen et al., 2024) |
| Antidiabetic | Hydrolysis (Alcalase) | Quinoa | Protein Hydrolysate (<1000 Da) | - | Enzyme inhibition assay (α-amylase/glucosidase) | α-amylase inhibition: IC₅₀ = 3.66 mg/mL; α-glucosidase inhibition: IC₅₀ = 3.31 mg/mL | (Gan et al., 2025) |
| Antidiabetic | Hydrolysis (Trypsin) | Quinoa | Protein Isolate (>3 000 Da) | - | Enzyme inhibition assay (α-glucosidase) | Hydrolysate at 0.5 h; 44.79% of inhibition | (Abbasi et al., 2022) |
| Antidiabetic | Hydrolysis (Thermolysin)  +  Synthesis | Quinoa | Synthetic Peptide SPH | - | Enzyme inhibition assay (DPP-IV) | Peptide SPH with IC_50_ = 0.54 mg/mL | (Chirinos, Escobar-Mendoza, et al., 2023) |
| Antidiabetic | Hydrolysis (Chymotrypsin) | Quinoa | Hydrolysate (6 h) | - | Enzyme inhibition assay (DPP-IV) | IC_50_ = 0.72 mg/mL | (Mudgil et al., 2020) |
| Antidiabetic | Germination  +  Hydrolysis (Pepsin-Trypsin)  +  Ultrafiltration (<1000 Da) | Quinoa | Fraction <1000 Da | Cells (Caco-2) | Enzyme inhibition assay (DPP-IV) | IC₅₀ = 2.20 mg/mL; peptide IPI identified; activity confirmed *in situ* | (You et al., 2022) |
| Anti-hypercholesterolemic (CEase) | Hydrolysis (Chymotrypsin) | Quinoa | Hydrolysate (2 h) | - | Enzyme inhibition assay (cholesterol esterase) | Hydrolysate: IC₅₀ = 0.51 mg/mL vs intact protein (IC₅₀ = 1.01 mg/mL) | (Ajayi et al., 2023) |
| Anti-hypercholesterolemic (Lipase) | Hydrolysis (Chymotrypsin) | Quinoa | Hydrolysate (6 h) | - | Enzyme inhibition assay (pancreatic lipase) | IC₅₀ = 0.78 mg/mL; peptides FSAGGLP, QPHPGLGALCAAPPST | (Ajayi et al., 2023) |
| Antioxidant | Hydrolysis (Neutrase) +  Ultrafiltration  +  SEC | Quinoa | Peptide Fraction V-SEC (<3000 Da) | - | ABTS radical scavenging assay | Purified fraction with 3784.85 µmol TE/g protein (2.3-fold increase vs crude) or hydrolyzed protein of 1667.38 µmol TE/g protein | (Cisneros-Yupanqui et al., 2022) |
| Antioxidant | Ultrasound (400 W)  +  Hydrolysis  (Alcalase) | Quinoa | Protein Isolate | - | DPPH radical scavenging assay | Ultrasound: 75% scavenging (6 mg/mL) vs thermal treatment ~55% | (X. Li et al., 2018) |
| Antioxidant | Fermentation (*L. plantarum* T0A10) | Quinoa | Purified fraction (qf2) | Cells (Human Keratinocytes) | Cell viability assay/ ROS assay | Purified fraction (0.1 mg/ml) protected cells from oxidative stress of 68.7%; activity comparable to α-tocopherol (500 to 1000 μmol/L). | (Rizzello et al., 2017) |
| Antioxidant / Cytoprotective | Sequential Hydrolysis (Alkaline protease  +  Trypsin) | Quinoa | Synthetic Peptide (GPGGGGKGEMF) | Cells (RAW264.7) | ROS assay + western blot | Reduced ROS; activation of Keap1–Nrf2 pathway | (Xiao et al., 2025) |
| Antioxidant | Hydrolysis (Chymotrypsin) | Quinoa | Protein Isolate | - | ABTS radical scavenging/ DPPH radical scavenging assay | Hydrolysate at 6 h; 498.6 µmol TE/mg protein (ABTS) and 1188.0 µmol TE/mg protein (DPPH) | (Mudgil et al., 2019) |
| Antioxidant | Hydrolysis  (Alcalase, compound protease, neutral protease, flavourzyme, papain) | Quinoa | Protein Hydrolysate | - | ABTS radical scavenging activity/ Hydroxyl radical scavenging activity | Peptides IC_50_=0.57 mg/mL (ABTS) and IC_50_ = 1.95 mg/mL (hydroxyl radicals) | (Gu et al., 2024) |
| Antioxidant | Hydrolysis  (Alcalase and trypsin) | Quinoa | Protein Isolate | - | ABTS radical scavenging assay | Hydrolysate (<10 kDa) at 0.5 h: IC_50_ = 0.804 mg/mL (trypsin) and IC_50_ = 8.791 mg/mL (alcalase) | (Abbasi et al., 2022) |
| Anti-inflammatory | Enzymatic Hydrolysis (Papain, Pepsin, Pancreatin) | Quinoa | Hydrolysate protein | Cells (RAW264.7) | No production assay | NO inhibition; no significant differences vs control | (Shi et al., 2019) |
| Antioxidant | Solid-State Fermentation (*R. oligosporus*) | Quinoa (Black) | Fermented seeds | - | ABTS radical scavenging assay | 4-day fermentation increased ABTS scavenging activity to up 185.06 µmol TE/g dwb. Raw seed 49.20 mol TE/g dwb. | (Starzyńska-Janiszewska et al., 2019) |
| Antioxidant | Fermentation (*R. oligosporus*) | Quinoa (Black) | Fermented seeds | - | ABTS assay | Fermentation (6 days) increased activity to 227.71 µmol Trolox/g dwb | (Starzyńska-Janiszewska et al., 2019) |
| ACE Inhibitory | Hydrolysis (Thermolysin) | Red Quinoa | Seed Protein | - | Enzyme inhibition assay (ACE) | 77.67% at 16 h; IC₅₀ = 0.20 mg/mL | (Suleman et al., 2024) |

EDUF, Electrodialysis with Ultrafiltration Membranes; HHP, High Hydrostatic Pressure; SEC, Size Exclusion Chromatography; RP-FPLC, Reversed-Phase Fast Protein Liquid Chromatography; RP-HPLC, Reversed-Phase High Performance Liquid Chromatography; MIC, Minimum Inhibitory Concentration; IC50, Half maximal inhibitory concentration; NO, Nitric oxide.

**Supplementary Table S3.** Extended compilation of in vitro bioactivities reported for peptides obtained after simulated gastrointestinal digestion of Andean grains.

| **Test** | **Treatment** | **Bioactive Activity** | **Andean Grain** | **Source / Fraction** | **Model** | **Assessment Method** | **Main Result of bioactivity** | **Source** |
| --- | --- | --- | --- | --- | --- | --- | --- | --- |
| In vitro | SGID (pepsin-pancreatin) | Antioxidant | Amaranth | Digest of protein isolate (Id) | – | ORAC assay | IC₅₀ =~ 0.024 mg/mL; ~5-fold higher activity vs undigested protein isolate | (Orsini et al., 2015) |
| In vitro | SGID | Antioxidant | Amaranth | Digest of isolate (Id) | - | ORAC assay | IC_50_ = ~0.024 mg/mL; more than alcalase hydrolysis | (Orsini et al., 2015) |
| In vitro | SGID (pepsin-pancreatin) | Antioxidant | Amaranth | Digest of Protein Isolate (Id) | – | Electron Spin Resonance (ESR) | >70% hydroxyl radical scavenging at 2 mg/mL, proving more effective than alcalase hydrolysates | (Orsini et al., 2015) |
| In vitro | SGID (pepsin-pancreatin) + Ultrafiltration (3000 Da) | Hypocholesterolemic | Amaranth | Permeate  (3000 Da) | – | Enzyme inhibition assay (HMG-CoA reductase) | Peptides (GGV, IVG) inhibited the key enzyme in cholesterol synthesis. | (Soares et al., 2015) |
| In silico | SGID  (pepsin-trypsin-chymotrypsin) | Antidiabetic | Amaranth; Quinoa | Theoretical Fragments | – | Molecular docking | 52 peptides identified (e.g. IW, PW) with affinity for DPP-IV. | (Valenzuela Zamudio et al., 2022) |
| In vitro | SGID (pepsin-pancreatin) | Antimicrobial | Cañihua | Glutelin digest (4 h) | Microorganisms  (*E. coli*) | Bacterial growth inhibition assay | 95% growth inhibition (*E. coli*) | (Moscoso-Mujica et al., 2021) |
| In vitro | SGID (INFOGEST) + Ultrafiltration (>5000 Da) | Cellular antioxidant | Cañihua | Intestinal digest (>5000 Da) | Cells  (Caco-2) | Cellular Antioxidant Activity (CAA) | 85.55% cellular protection | (Serena-Romero et al., 2023) |
| In vitro | Sequential Hydrolysis (pepsin-pancreatin) + Alcalase) + Ultrafiltration (<3000 Da) + Nanoencapsulation | Cytoprotective (Indirect antimicrobial) | Lupin | Encapsulated peptide fraction | Bacteria (*E. coli*) | Viable count assay (CFU) | 43.25% increase in bacterial survival under oxidative stress | (Intiquilla et al., 2022) |
| In vitro | Sequential Hydrolysis (pepsin-pancreatin) + Alcalase) + Ultrafiltration (<3000 Da) | Antioxidant | Lupin | Low molecular weight fraction | – | ORAC assay/ ABTS assay/ iron chelation assay | ORAC: 5.02 μmol TE/mg; iron chelation: 96.39% | (Intiquilla et al., 2022) |
| In vitro | SGID  (Pepsin) | Anti-adipogenesis | Quinoa | Hydrolysate (120 min) | Cells  (3T3-L1) | Lipid Accumulation assay | IC_50_ = 786.58 µg/mL; inhibited adipogenesis | (Shi et al., 2019) |
| In vitro | SGID  (pepsin-pancreatin) | Anticancer (Cytotoxicity) | Quinoa | Intestinal Digest (60 min) | Cells  (Caco-2) | MTT Assay (IC_50_) | IC₅₀ = 0.19 mg/mL (fraction >5000 Da) | (Vilcacundo et al., 2018) |
| In vitro | SGID  (pepsin-pancreatin) +  Ultrafiltration (<5000 Da / >5000 Da) | Antidiabetic | Quinoa | Intestinal Digest | – | Enzyme inhibition assay (DPP-IV) | IC_50_ = 0.25 mg/mL; 10-fold more potent than the gastric phase | (Vilcacundo et al., 2017) |
| In vitro | SGID  (pepsin-pancreatin) | Antidiabetic | Quinoa | Intestinal Digest (120 min) | – | Enzyme inhibition assay (α-amylase) | IC_50_ = 0.19 mg/mL | (Vilcacundo et al., 2017) |
| In vitro | SGID (pepsin-pancreatin) + Purification | Antihypertensive | Quinoa | Purified Fraction (C4) | – | Enzyme inhibition assay (ACE) | IC₅₀ = 0.05 mg/mL; peptides EHPEPR, NWEPLPR, NIEPRF | (Guo, Hao, et al., 2020) |
| In vitro | Germination (12 h) + SGID (Pepsin -trypsin) | Antioxidant (Mechanism) | Quinoa | Identified peptides (FGDL, FGGL) | Molecular docking | Docking with (Keap1-Kelch domain) | Strong binding to Keap1; potential activation of Nrf2 pathway | (He et al., 2025) |
| In vitro | Enzymatic Hydrolysis (Alcalase) + SGID (Pepsin-pancreatin) | Antioxidant (Stability) | Quinoa | Digested hydrolysates | – | ABTS radical scavenging assay/ Hydroxyl radical scavenging activity | 93.8% activity retained after digestion (<1000 Da peptides) | (Gu et al., 2024) |
| In vitro | SGID (pepsin-pancreatin) | Antioxidant / Digestibility | Quinoa | Globulin Digest | – | DPPH assay/ Enzyme inhibition assay (ACE) | Digested globulin (24 h): increased antioxidant activity and ACE inhibition after digestion | (Tavano et al., 2022) |
| In vitro | Germination (12 h) + SGID (Pepsin + Trypsin) + Centrifugation | Cytoprotective / Antioxidant | Quinoa | Gastrointestinal digest | Cells (HepG2) | Cell viability assay (CCK-8)/antioxidant enzyme activity (SOD/CAT) | Cell viability: 88.39%; increased SOD and CAT activity | (He et al., 2025) |
| In vitro | SGID (Static Model) + Purification (>6000 Da) | Hypoallergenicity (Safety) | Quinoa | Flour and Protein Isolate digests | Humans sera (allergic patients) | Immunoblotting assay (IgE binding, Western blot) | Resistant peptides showed no IgE reactivity with sera from patients allergic to wheat/cereals, confirming safety | (Romano et al., 2019) |
| In vitro | Germination + Hydrolysis (pepsin-trypsin) + Ultrafiltration (<1000 Da) | Antidiabetic | Quinoa | Fraction <1000 Da | Cells  (Caco-2) | Enzyme inhibition assay (DPP-IV) | Germinated IC_50_ = 2.20 mg/mL; Peptide IPI identified.  Small peptides exhibited strong in situ DPP-IV inhibition. | (You et al., 2022) |
| In vitro | SGID (pepsin-pancreatin) + pre-hydrolyzed proteins (Neutrase) | Antioxidant | Quinoa | Digested Hydrolysates | - | ABTS radical cation scavenging assay | No significant changes after digestion; ~ 1.54 µmol TE/mg vs before 1.50 µmol TE/mg | (Chirinos et al., 2020) |

SGID, Simulated Gastrointestinal Digestion; ACE; Angiotensin-Converting Enzyme; DPPH, 2,2-diphenyl-1-picrylhydrazyl; ABTS, 2,2′-azinobis (3-ethylbenzothiazoline-6-sulfonic acid); GI, Gastrointestinal; ORAC, Oxygen Radical Absorbance Capacity; SOD, Superoxide Dismutase; CAT, Catalase; HepG2, Human Liver Cancer Cell Line**.**
